# MicroRNA Profile in CD8+ T-Lymphocytes from HIV-Infected Individuals: Relationship with Antiviral Immune Response and Disease Progression

**DOI:** 10.1371/journal.pone.0155245

**Published:** 2016-05-12

**Authors:** Lander Egaña-Gorroño, Alberto C. Guardo, Manel E. Bargalló, Evarist Planet, Elisenda Vilaplana, Tuixent Escribà, Iñaki Pérez, Josep Maria Gatell, Felipe García, Mireia Arnedo, Montserrat Plana M, HIV Controllers Consortium of the AIDS Spanish Network

**Affiliations:** 1 Group of Genomics and Pharmacogenomics of HIV, AIDS Research Group, Institut d’Investigacions Biomèdiques August Pi i Sunyer (IDIBAPS), Hospital Clínic de Barcelona, Barcelona, Spain; 2 Immunopathology and Cellular Immunology, AIDS Research Group, Catalan project for the development of an HIV vaccine (HIVACAT), Institut d’Investigacions Biomèdiques August Pi i Sunyer (IDIBAPS), Hospital Clínic de Barcelona, Barcelona, Spain; 3 School of Life Sciences, Ecole Polytechnique Fédérale de Lausanne (EPFL), 1015 Lausanne, Switzerland; 4 Department of Systems Biology, University of Vic-Central University of Catalonia, Vic, Spain; 5 Department of Infectious Diseases, Hospital Clínic de Barcelona, University of Barcelona, Barcelona, Spain; Massachusetts General Hospital, UNITED STATES

## Abstract

**Background:**

The relationship between host microRNAs (miRNA), viral control and immune response has not yet been elucidated in the field of HIV. The aim of this study was to assess the differential miRNA profile in CD8+ T-cells between HIV-infected individuals who differ in terms of viral replication control and immune response.

**Methods:**

miRNA profile from resting and CD3/CD28-stimulated CD8+ T-cells from uninfected individuals (HIV-, n = 11), Elite Controllers (EC, n = 15), Viremic Controllers (VC, n = 15), Viremic Progressors (VP, n = 13) and HIV-infected patients on therapy (ART, n = 14) was assessed using Affymetrix miRNA 3.1 arrays. After background correction, quantile normalization and median polish summarization, normalized data were fit to a linear model. The analysis comprised: resting samples between groups; stimulated samples between groups; and stimulated versus resting samples within each group. Enrichment analyses of the putative target genes were perfomed using bioinformatic algorithms.

**Results:**

A downregulated miRNA pattern was observed when resting samples from all infected groups were compared to HIV-. A miRNA downregulation was also observed when stimulated samples from EC, ART and HIV- groups were compared to VP, being hsa-miR-4492 the most downregulated. Although a preferential miRNA downregulation was observed when stimulated samples were compared to the respective resting samples, VP presented a differential miRNA expression pattern. In fact, hsa-miR-155 and hsa-miR-181a were downregulated in VP whereas in the other groups, either an upregulation or no differences were observed after stimulation, respectively. Overall, functional enrichment analysis revealed that the predicted target genes were involved in signal transduction pathways, metabolic regulation, apoptosis, and immune response.

**Conclusions:**

Resting CD8+ T-cells do not exhibit a differential miRNA expression between HIV-infected individuals but they do differ from non-infected individuals. Moreover, a specific miRNA pattern is present in stimulated CD8+ T-cells from VP which could reflect a detrimental pattern in terms of CD8+ T-cell immune response.

## Introduction

The majority of untreated HIV-infected individuals will develop AIDS within a few years, a process associated with chronic immune activation, persistent viral replication, and a severe decline of CD4+ T-cells [1]. However, a small group of HIV-infected individuals (5–15%) named long-term non progressors (LTNPs) control disease progression for >7 years in the absence of antiretroviral therapy (ART) [2]. Among this group, elite controllers (EC) represent a small group of individuals (<0.3%) that are capable of controlling viral replication (plasma viral load <50–75 RNA cp/ml) during years without ART [3,4]. Although the mechanisms responsible for the control of HIV replication in EC remain poorly defined, several host genetic factors linked to the quality of host innate and adaptive immune responses, as well as viral factors, have been proposed to be involved [4,5]. In fact, there is a large body of evidence indicating that the CD8+ T-cell-mediated immune response constitutes a major component of the protective immunity present in EC [6]. It has been shown that EC develop polyfunctional and potent memory T-cell response that is likely the underlying mechanism for the control of viral replication and disease progression in the absence of ART [7], since the persistence of functional memory T-cells represents the basis for a long-lasting protection after exposure to pathogens [8,9]. Understanding the molecular mechanisms involved in the immune response capable of controlling HIV replication and disease progression is a priority area of study in the field of HIV immunopathogenesis.

The discovery of a growing class of evolutionarily conserved small RNAs, termed microRNAs (miRNAs), has opened a new field of research and revealed the possibility to identify plausible miRNA that regulate a wide variety of biological processes including the immune response to viruses [10]. miRNAs correspond to 20–25 nucleotide-long non-coding RNAs that modulate gene expression through base pairing of the miRNA seed sequence to it’s target mRNA (usually located within the 3’UTR) [11]. This interaction leads to either translational repression or mRNA cleavage thereby inhibiting the final target protein production [12]. During virus infection and its replication, host and viral RNAs and miRNAs interact in various ways, mutually regulating their levels and translational competence [13]. Several reports on the differential expression of host and viral miRNAs and their roles in HIV infection have been published [14–18]. While several viral miRNAs can target critical host factors various interactions between HIV and cellular miRNAs have been described, suggesting that an altered miRNA profile expression could contribute to the pathogenesis of HIV and latency in primary CD4+ T-lymphocytes.

Differential miRNA profiles between HIV-infected individuals at different stages of disease progression could reveal a dysregulated miRNA pattern with a prognostic and diagnostic value for HIV-1 therapies. Furthermore, a differential regulation of miRNAs in CD8+ T-lymphocytes could help us to better understand the underlying mechanisms of an effective HIV antiviral response that controls viral replication and may predict the status of disease progression. Thus, the aim of this study was to compare the miRNA expression profile in CD8+ T-cells from HIV-infected individuals and to evaluate whether those differences could be associated with the control of viral replication and the effective antiviral immune response and thus, influence the future progression of HIV infection.

## Materials and Methods

### Study population

Samples were obtained from HIV-1-infected patients followed up at the HIV Unit of the Hospital Clinic of Barcelona (Barcelona, Spain). Samples of non-HIV infected donors, as a control group, were also obtained from the Blood and Tissue Bank (BST, Barcelona, Spain). Ethical committee approval and written informed consent from all subjects, in accordance with the Declaration of Helsinki, were obtained prior to study initiation. The study was approved by the institution ethical committee: Comitè Ètic d'Investigació Clínica, Hospital Clinic, Barcelona, Spain (Protocol approval numbers: 2012/7506). Sixty-eight individuals, classified in 5 groups, were included in the study: HIV-negative individuals (HIV-; n = 11), Elite Controllers (EC; n = 15; viral load <50 cp/ml for at least more than one year of follow-up in the absence of ART), Viremic Controllers (VC; n = 15; viral load <2000 cp/ml for at least more than one year of follow-up in the absence of ART), Viremic Progressors (VP; n = 13; viral load >5000 cp/ml for more than one year of follow-up in the absence of ART) and HIV-infected patients under antiretroviral treatment (ART; n = 14; viral load <50 cp/ml for more than one year of follow-up). Moreover, in order to have an homogeneous population and to avoid any influence of a severe immunodeficiency, all the participants had a current CD4+ T-cell count >450 cells/mm^3^. Medians were used to show central tendencies and interquartile ranges (IQR = upper quartile Q3-lower quartile Q1) were calculated as measures of variability and statistical dispersion in each group.

### Immune-separation of CD8+ T-lymphocytes

Peripheral blood mononuclear cells (PBMCs) were either isolated from fresh blood by Ficoll-Hypaque gradient centrifugation or used after thawing. PBMCs (20x10^6^ cells) were cultured in RPMI medium containing 10% FBS and 2% gentamycin. CD8+ T-lymphocytes were isolated for RNA extraction after a positive selection with monoclonal antibodies conjugated to microbeads AutoMACS (Miltenyi Biotec, Bergisch Gladbach, Germany). In order to avoid major natural killer (NK) cell contamination, a positive immunomagnetic isolation with CD56+ Miltenyi AutoMACS microbeads was performed followed by a positive selection with CD8+ Miltenyi AutoMACS microbeads. The cell suspension was loaded onto a column and placed in the magnetic field of a MACS Separator (Miltenyi Biotec, Bergisch Gladbach, Germany). The magnetically labeled CD8+ T-cells were retained while the unlabelled cells run through. After removing the column from the magnetic field, the magnetically retained CD8+ T-cells were eluted as the positively selected cell fraction. Flow cytometry analysis of cell purity showed that, the final isolated population was enriched by more than 85–90% of CD8+ T lymphocytes and contained <5% of CD3- CD56+ NK cells. Cell pellets were generated from CD8+ T-cells either resting or activated using 10 ng/ml of CD3-OKT3 (ORTHO biotech Products, L.P, Raritan, NJ, USA) and 400 μg/ml of CD28 (kindly provided by Dr. R. Vilella; Laboratory of Immunology, Hospital Clinic, Barcelona, Spain) during three days.

### RNA isolation and quality control

Total RNA (enriched for small non-coding RNAs) was isolated according to manufacturer’s instructions using the ReliaPrep RNA Cell miniprep system kit (Promega, Fitchburg, WI, USA). RNA concentration was calculated by fluorometric quantitation using Qubit (Life Technologies, Carlsbad, CA, USA). RNA integrity of each sample was evaluated using RNA 6000 Nano LabChips on an Agilent 2100 Bioanalyzer (Agilent Technologies, Santa Clara, CA, USA). All chips were prepared according to the manufacturer’s instructions at the Functional Genomics Core of the Institute for Research in Biomedicine (IRB, Barcelona, Spain). Total RNA degradation was evaluated by reviewing the electropherograms and the RNA integrity number (RIN) of each sample. Only samples with preserved 18S and 28S peaks and RIN values greater than 7 were selected for miRNA profile analysis.

### miRNA profiling using Affymetrix miRNA 3.1 strip arrays

miRNA expression profiles of resting and activated CD8+ T-lymphocytes were generated using the Affymetrix miRNA 3.1 strip arrays (Affymetrix, Santa Clara, CA, USA) at the Functional Genomics Core of the Institute for Research in Biomedicine (IRB, Barcelona, Spain). All procedures were carried out according to the manufacturer's protocols. Briefly, 1μg of each total RNA sample was poly-A tailed and the proprietary biotin-labeled dendramer molecule was joined to the 3´-end using the FlashTag Biotin RNA Labeling Kit (Genisphere, Hatfield, PA, USA). Labeled samples were hybridized to the miRNA array at 48°C for 16 h and then washed and stained with a Streptavidin-PE solution prior to imaging. Chips were scanned with a GeneChip scanner 3000 G7 (Affymetrix, Santa Clara, CA, USA). The Affymetrix miRNA 3.1 strip arrays contain 19.724 probe sets and covers 153 organisms, including humans. The strip array miRNA probes were derived from the Sanger miRBase miRNA database v17 release (http://microrna.sanger.ac.uk) and include a total of 5.607 human miRNAs: 1.733 human mature miRNA (hsa-miR) probe sets, 2.216 human small nucleolar RNAs (snoRNA) and small cajal body-specific RNAs (scaRNA) probe sets and 1.658 human pre-miRNA (hsa-mir) probe sets.

### Accessibility of array data

Raw data and corrected, quantile and median polished normalized data from Affymetrix miRNA 3.1 strip arrays were deposited with the Gene Expression Omnibus (GEO, [19]) and are accessible at Series record number GSE71650.

### Array data analysis

All microarray statistical analyses were performed using Bioconductor (http://www.bioconductor.org) [20]. After background correction, quantile normalization and median polish summarization the normalized data were fit to a linear model as implemented in bioconductor’s affy package [21]. The effects of gender and having hybridized the arrays on different dates were corrected with an Analysis Of Variance (ANOVA) adjustment. Moderated t-tests (as implemented in the Bioconductor limma package) were performed to obtain p-values [22]. Benjamini-Hochberg correction test was applied as an estimated false discovery rate (FDR) of 5%, controlling for the expected proportion of incorrectly rejected null hypotheses [23]. A microRNA was considered to be differentially expressed with a non-adjusted p-value <0.05 and an absolute fold change value >±1.5. A differential expression of <5 miRNAs was considered as a non-differential microRNA profile. The analysis was classified according to: a) resting samples between groups; b) stimulated samples between groups; and c) stimulated versus resting samples within each group. Graphs were represented as fold change (mean) using GraphPad Prism 5.0 (GraphPad Software, La Jolla, CA, USA).

### Target gene prediction and functional enrichment analysis

The gene target prediction for the differentially expressed miRNA in each comparison was performed using miRWalk v.2.0 (http://www.umm.uni-heidelberg.de/apps/zmf/mirwalk/) online software [24]. Significantly predicted target genes were selected fixing a p-value <0.01. The enrichment analyses (EA) were performed using DAVID bioinformatics resources [25,26] on the basis of Gene Onthology-Biological Process (GO-BP) terms to each list of miRNA predicted target genes. Whole human genome was used as background for the analysis. The significance of gene-term enrichment was examined using the Expression Analysis Systematic Explorer (EASE) score and a statistical threshold fixed at p-value <0.05 [27].

### Validation of miRNA expression profiles

Due to limiting amount of RNA, 4 miRNAs of interest (hsa-miR-155-5p [ID 002623], hsa-miR-149-3p [ID 002164], hsa-miR-4484 [ID 464264_mat], hsa-miR-4485 [ID 472953_mat]) were re-assessed through individual RT-qPCR assay (Applied Biosystems, Foster City, CA, USA). The analysis included the validation of the differential expressions observed in the miRNA profiling analysis, in the same study population, for the mentioned miRNAs. RNA (10 ng) was reverse transcribed in 15 μl according to manufacturer’s recommendations using TaqMan miRNA reverse transcription kit (Applied Biosystems, Foster City, CA, USA). miRNA expression assays were carried out using TaqMan primers and probes (Applied Biosystems, Foster City, CA, USA) for endogenous control small RNAs RNU44 (ID 001094) and RNU48 (ID 001006) and target miRNAs. Relative quantifications (RQ) were performed using the Applied Biosystems 7900HT Fast Real-time PCR system. Reaction volumes contained: 7.67 μl of water, 1 μl of TaqMan primer/probe mix for target or endogenous control small RNA, 10 μl of Universal master mix (Applied Biosystems, Foster City, CA, USA) and 1.33 μl of cDNA at a final concentration of 10 ng. Thermocycler conditions were as follows: 95°C hot-start for 10 min, followed by 40 cycles of 95°C for 15 s and 60°C for 1 min. Raw Ct values were exported from the SDS software v.2.3 to the RQ Manager v1.2 softwear (Applied Biosystems, Foster City, CA, USA) for ΔCt (ΔCt = Ct_target miRNA_—mean Ct_endogenous small RNAs_) value determination as the normalization method. Expression levels of the individual miRNAs relative to the mean expression of the small RNAs were calculated as 2^-ΔCt^. Oneway analysis of variance (ANOVA) tests were performed for global intergroup comparisons and Turkey post-hoc tests for pair comparisons; and two-way ANOVA tests were performed for global intragroup comparisons and Bonferroni post-tests for replicate-means comparisons using GraphPad Prism 5.0 (GraphPad Software, La Jolla, CA, USA).

## Reults

### Characteristics of the study participants

Characteristics of the study participants are shown in Table 1 (anonymous and confidential data for HIV-). Patients in every group were predominantly men who had sex with men, between 34 and 56 years old. For more than a half of them median CD4+ T-cell count was over 600 cells/mm^3^ in all groups. As expected, statistically significant differences regarding NADIR CD4+ T-cell count, baseline and current viral load, were observed between the groups. At the time of inclusion none of them was coinfected by hepatitis C/B virus (HCV/HBV). After seven years of follow-up all the participants from the EC and the VC group maintained viral load <50 cp/ml and <2000 cp/ml, respectively, and CD4+ cell count >450 cells/mm^3^. A heterogeneous distribution of HLA-B57*01 was observed and none of them showed the CCR5 delta-32 gene deletion (data not shown).

**Table 1:** Baseline characteristics of the study participants.

Participant characterictics	VC (n = 15)	VP (n = 13)	EC (n = 15)	ART (n = 14)	p-value
Age, years^#^	41 (13.5)	37.5 (7.5)	45.5 (20)	43 (16)	NS
Men/women, n men (%)	12 (75)	13 (93)	11 (61)	14 (82)	NS
Presumed mode of HIV transmission, n (%)					
*MSM*	13 (81.3)	12 (85.7)	9 (50)	11 (64.7)	NS
*Heterosexual*	2 (12.5)	1 (7.1)	4 (22.2)	3 (17.6)	NS
*IVDU*	1 (6.2)	0 (0)	3 (16.6)	1 (5.9)	NS
*Other/unknown*	0 (0)	1 (7.1)	2 (11.2)	2 (11.8)	NS
CD4+ T-cell count (cells/ml)^#^	654 (539–846)	626 (534–717)	771 (655–1103)	840 (579–945)	NS (0.09)
Nadir CD4+ T-cell count (cells/ml)^#^	505 (436–572)	445 (361–606)	533 (456–762)	333 (271–471)	**
CD8+ T-cell count (cells/ml)^#^	1182 (787–1624)	1028 (802–1377)	896 (644–1311)	754 (583–1200)	NS (0.07)
Plasma viral load (log) ^&^	2.9 (0.2)	4.3 (0.1)	1.8 (0.1)	1.6 (0)	***
Baseline viral load (log)^&^	2.8 (0.2)	3.8 (0.2)	1.6 (0)	3.6 (0.2)	***
Time since HIV diagnosis, years^#^	6 (2–12.2)	4.5 (3.7–9)	10 (4–12.5)	9 (5–13)	NS
Time of exposure to ART, years^#^	N/A	N/A	N/A	5 (9.5)	N/A

VP, viremic progressors; EC, elite controllers; ART, patients on antiretroviral therapy; VC, viremic controllers; IVDU, intravenous drug users; MSM, men who had sex with men; N/A, not applicable;

^#^median [IQR, P25-P75];

^&^mean (SEM); p-value, ANOVA or χ2 Test were performed where corresponds, all P values of statistically significant differences are indicated in bold (* p< 0.05; ** p< 0.01; *** p< 0.001). NS, non-significant.

### Differential miRNA profile between resting CD8+ T-cells from the different groups

A downregulated miRNA pattern was observed when resting samples from all infected groups were compared to HIV-. The expression analysis showed 16, 26, 30 and 52 and downregulated miRNAs when resting samples from VP, EC, VC and ART were compared to HIV-, respectively (S1 Table). When the expression profile of resting samples from VP, EC, VC and ART were compared to HIV-, eleven miRNAs were ubiquitously downregulated in the four comparisons: hsa-mir-1469, hsa-mir-4507, hsa-mir-663a, hsa-miR-1469, hsa-miR-1587, hsa-miR-4505, hsa-miR-4507, hsa-miR-4651, hsa-miR-4674, hsa-miR-4734, hsa-miR-663a.

**Fig 1:**
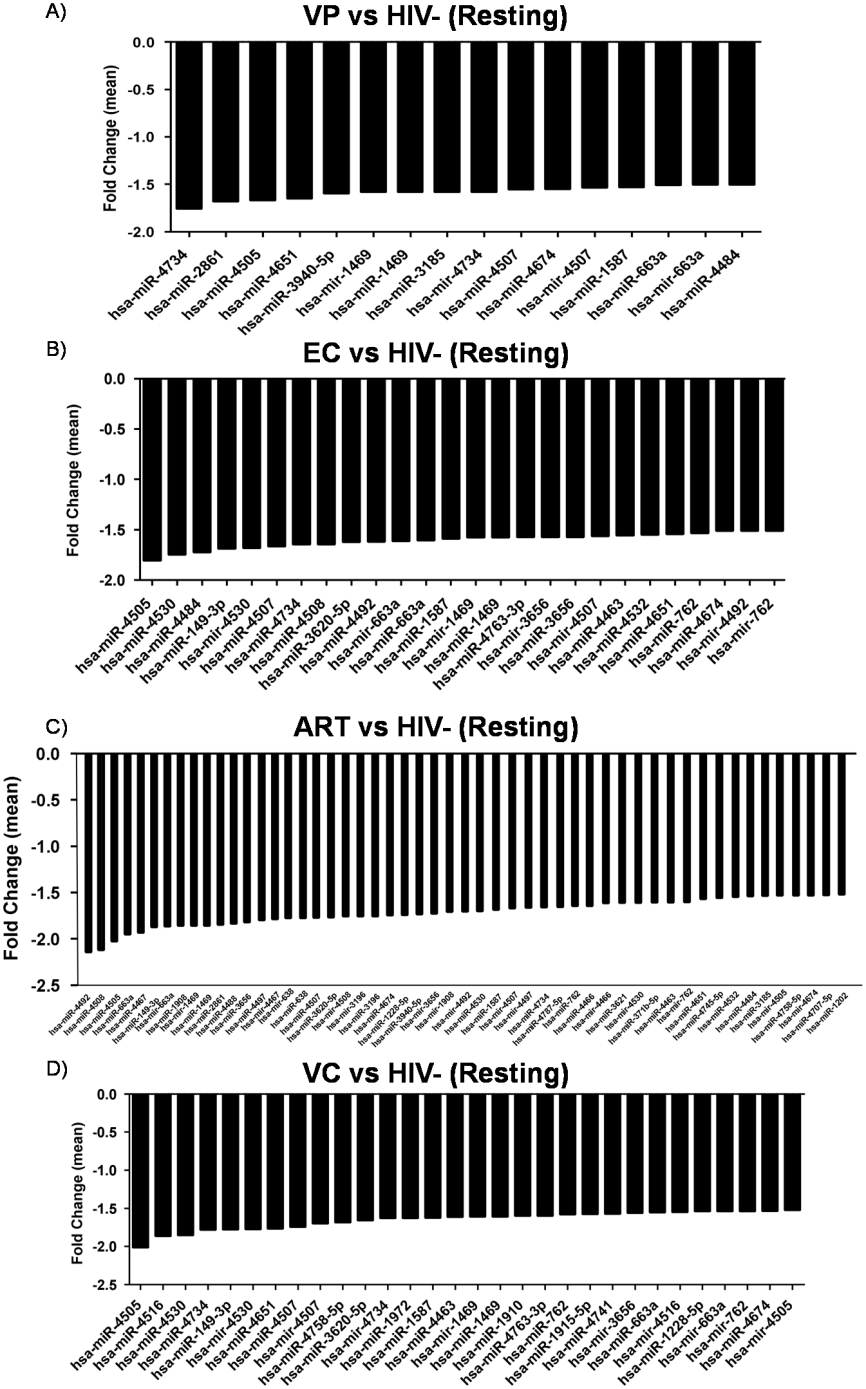
Differential miRNA profile between resting CD8+ T-cells. Graphs represent mean fold change of the differential miRNA expression between resting CD8+ T-cells from: A) VP vs HIV-; B) EC vs HIV-; C) ART vs HIV-: and D) VC vs HIV-. A fold change value of >±1.5 was considered. *VP*, *viremic progressors; EC*, *elite controllers; ART*, *patients on antiretroviral therapy; HIV-*, *uninfected donors; VC*, *viremic controllers*.

The most downregulated specific miRNAs in each comparison were: hsa-miR-4734 in VP vs HIV-, hsa-miR-4505 in EC vs HIV- and VC vs HIV-, and both hsa-miR-4492 and hsa-miR-4508 in ART vs HIV- (S2 Table, Fig 1).

None of the assessed miRNAs was differentially expressed when comparing VP versus EC, VP versus VC, EC versus ART, EC versus VC and ART versus VC (S2 Table, Fig 1). Out of all the miRNAs assessed, only hsa-miR-4492 was upregulated when VP were compared to the ART group.

### Differential miRNA profile between stimulated CD8+ T-cells from the different groups

**Fig 2:**
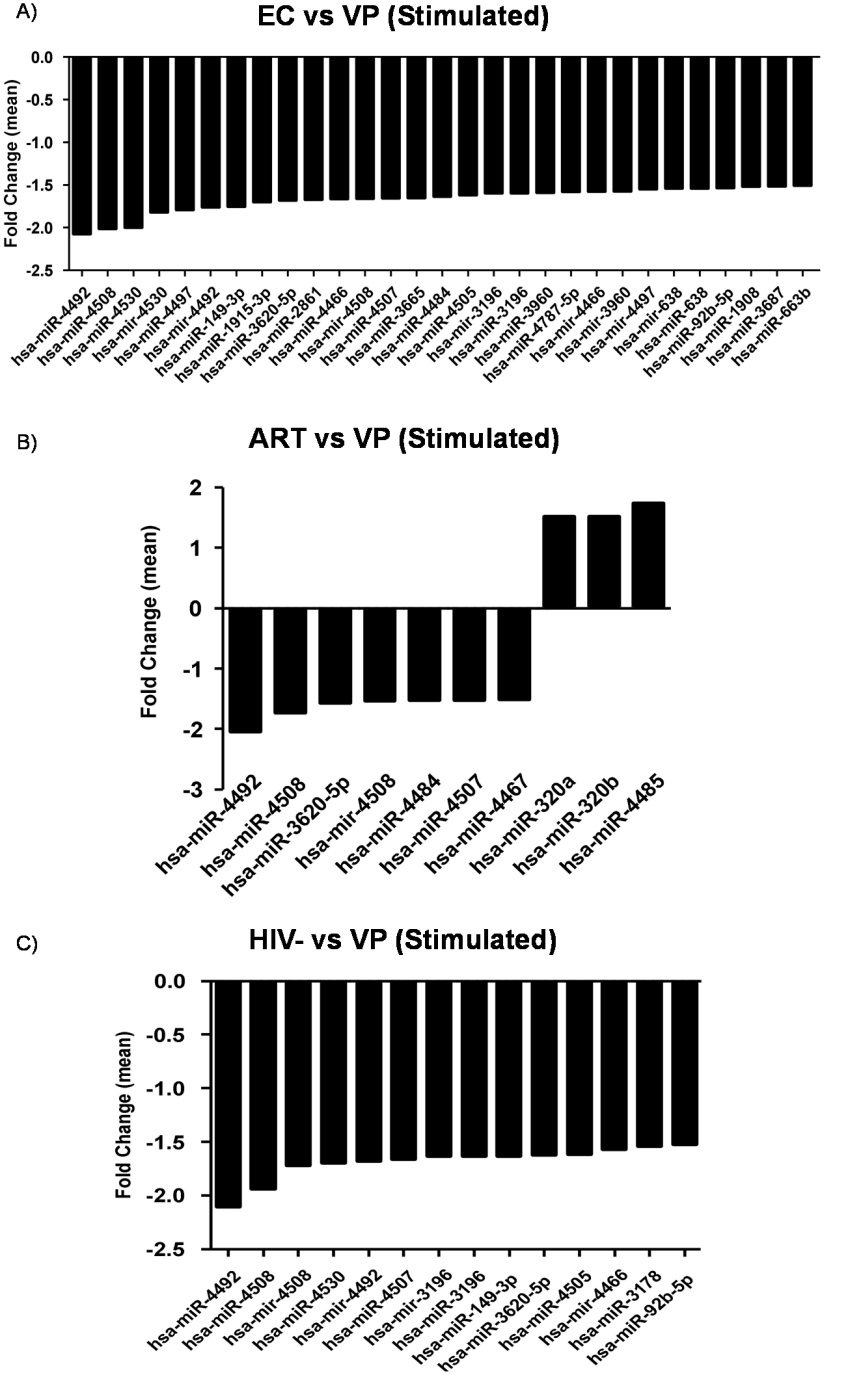
Differential miRNA profile between stimulated CD8+ T-cells. Graphs represent mean fold change of the differential miRNA expression between stimulated CD8+ T-cells from: A) EC vs VP; B) ART vs VP; and C) HIV- vs VP. A fold change value of >±1.5 was considered. *VP*, *viremic progressors; EC*, *elite controllers; ART*, *patients on antiretroviral therapy; HIV-*, *uninfected donors; VC*, *viremic controllers*.

The expression analysis showed 29, 10 and 14 differentially expressed miRNAs when stimulated samples from EC, ART and VIH- were compared to VP, respectively. As previously observed when comparing resting samples, a preferential miRNA downregulation was observed when comparing stimulated samples, except for three miRNA (hsa-miR-320a, hsa-miR-320b and hsa-miR-4485) that were upregulated when stimulated samples from ART were compared to the VP (S1 Table). No differential expression of miRNAs was observed when comparing VP versus VC, and ART versus HIV- (S3 Table, Fig 2).

When the expression profile of stimulated samples from EC, ART and HIV- were compared to VP, five miRNAs were ubiquitously downregulated in the three comparisons: hsa-mir-4508, hsa-miR-3620, hsa-miR-4492, hsa-miR-4507 y hsa-miR-4508.

The most downregulated specific miRNAs in each comparison was hsa-miR-4492 in EC vs VP, ART vs VP and HIV- vs VP (S3 Table, Fig 2).

### Differential miRNA profile between stimulated and resting CD8+ T-cells

**Fig 3:**
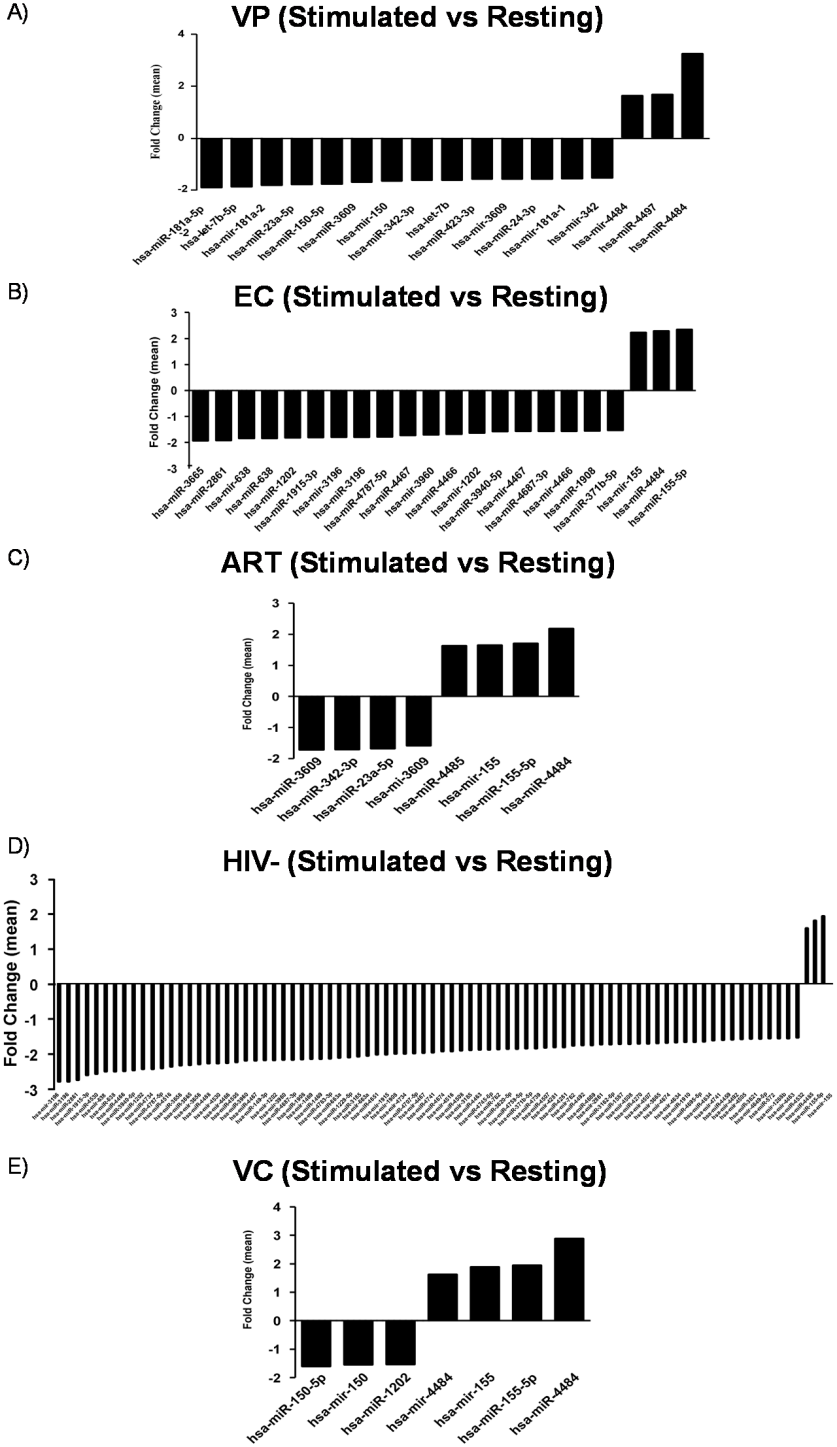
Differential miRNA profile between stimulated and resting CD8+ T-cells. Graphs represent mean fold change of the differential miRNA expression between stimulated and resting CD8+ T-cells from: A) VP; B) EC; C) ART; D) HIV-; and E) VC. A fold change value of >±1.5 was considered. *VP*, *viremic progressors; EC*, *elite controllers; ART*, *patients on antiretroviral therapy; HIV-*, *uninfected donors; VC*, *viremic controllers*.

A preferential miRNA downregulation was observed when stimulated CD8+ T-cell samples were compared to the respective resting samples. The expression analysis showed 17, 22, 7, 8 and 83 differentially expressed miRNAs when stimulated samples from VP, EC, VC, ART and HIV- and were compared to resting samples, respectively (S1 and S4 Tables, Fig 3).

Immature hsa-mir-155 and mature hsa-miR-155 were upregulated when resting CD8+ T-cells were stimulated in every group of study, except in VP. Moreover, hsa-miR-155 was one the most differentially expressed miRNA. Similarly, hsa-miR-4484 was one the most upregulated miRNA in every group of study, except in the non-infected group. The most downregulated miRNAs were hsa-miR-181a and hsa-let-7b-5p in VP, hsa-miR-3665 and hsa-miR-2861 in EC, hsa-miR-3609 and hsa-miR-342 in ART, hsa-mir/miR-3196 and hsa-miR-2861 in HIV- and hsa-mir/miR-150 and hsa-miR-1202 in VC (S4 Table, Fig 3).

### Target gene prediction of the differentially expressed miRNAs and functional analysis

The number of predicted gene targets for all the differentially expressed miRNAs in each comparison is shown in S5 Table.

**Fig 4:**
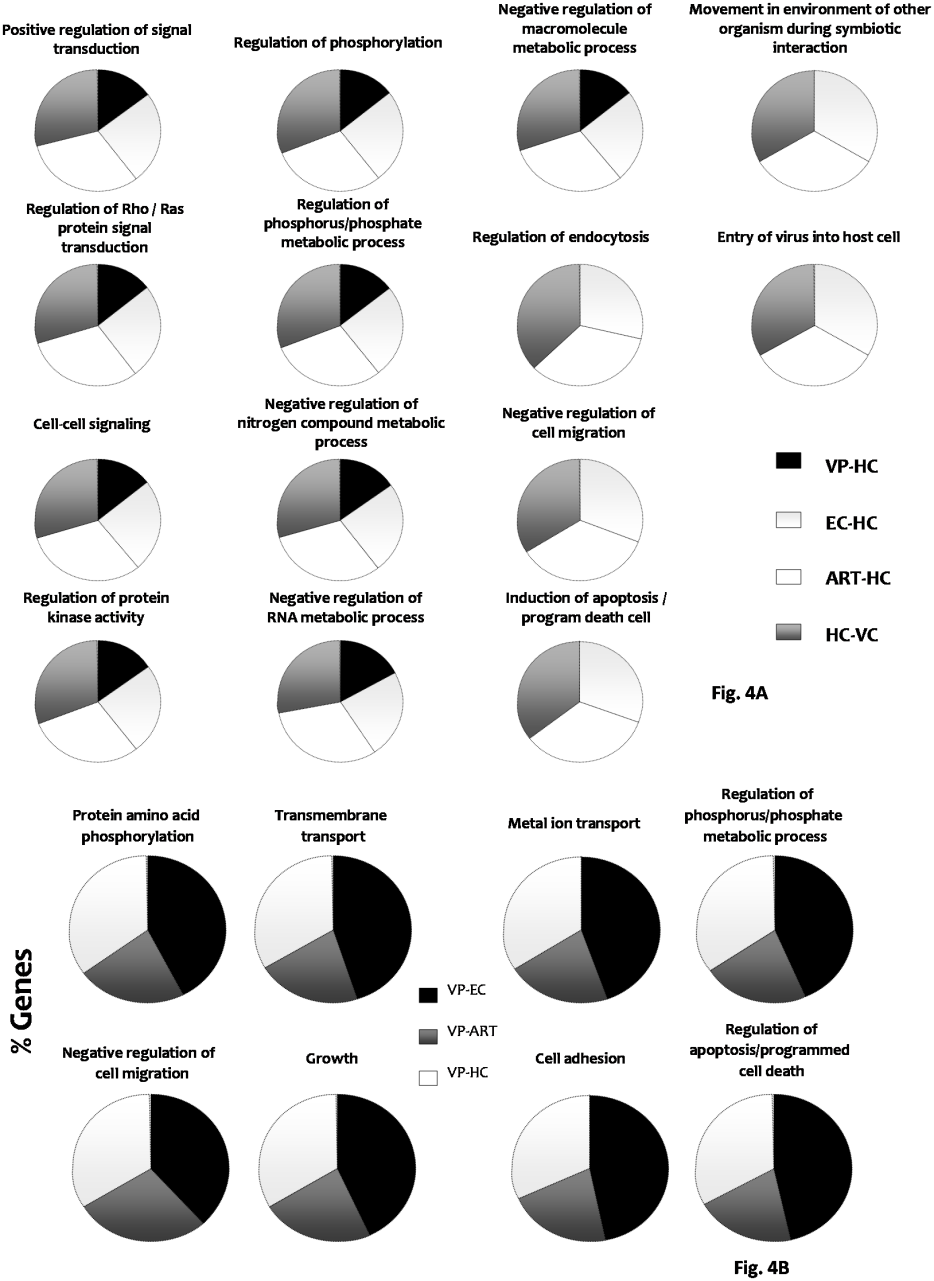
Predicted target genes for all the differentially expressed miRNAs. Graphs represent the percentage of predicted target genes grouped by pathways for the differential miRNA expression pattern in comparisons with resting CD8+ T cell samples (A) and with stimulated CD8+ T cell samples (B). *VP*, *viremic progressors; EC*, *elite controllers; ART*, *patients on antiretroviral therapy; HIV-*, *uninfected donors; VC*, *viremic controllers*.

Functional enrichment analysis revealed that mostly, predicted target gene pathways were involved in signal transduction, metabolic regulation, apoptosis and immune response and. Predicted gene targets involved in the regulation of signal transduction (Rho/Ras signalling, cell-cell signalling, protein kinase activity and phosphorylation activity) and metabolic regulation (phosphorus/phosphate metabolism, negative regulation of nitrogen compounds, RNA and macromolecule processes) were found when resting CD8+ T-cell samples from VP, EC, VC and ART were compared to HIV-. Target genes involved in apoptosis and in the regulation of immune response were also predicted for these comparisons (Fig 4A).

Regarding the differentially expressed miRNAs between stimulated CD8+ T-cells from EC, ART and HIV- vs VP, target gene analysis showed connections with protein phosphorylation and phosphate metabolic processes, membrane transport, apoptosis, cell adhesion, growth and migration pathways (Fig 4B).

Finally, a great number of genes involved in several pathways were predicted as targets for the differentially expressed miRNAs between stimulated CD8+ T-cells from HIV- compared to the respective resting samples (data not shown). However, these targets were not commonly predicted in any of the other four groups.

Of note, target genes involved in regulation of NF-kappa B cascade, DNA, nucleotide, lipid and catecholamine metabolism, cell death, apoptosis and homeostasis, differentiation of B cells, cell growth and leukocyte activation, exocytosis, degranulation and viral entry and infection, were only observed in EC. By contrast, predicted target genes for VP were connected to dephosphorylation, glycoprotein biosynthesis, IgG isotype switching, receptor-mediated endocytosis, cell adhesion, lymphocyte and T-cell differentiation, cell growth, and B cell activation (data not shown).

### Validation of miRNA expression profiles through individual RT-qPCR assays

In order to strengthen the expression patterns observed in the miRNA profiling analysis, mature hsa-miR-155-5p, -149-3p, -4484 and -4485 were re-assessed in the same samples from the study population through individual RT-qPCR assay (Table 1).

**Fig 5:**
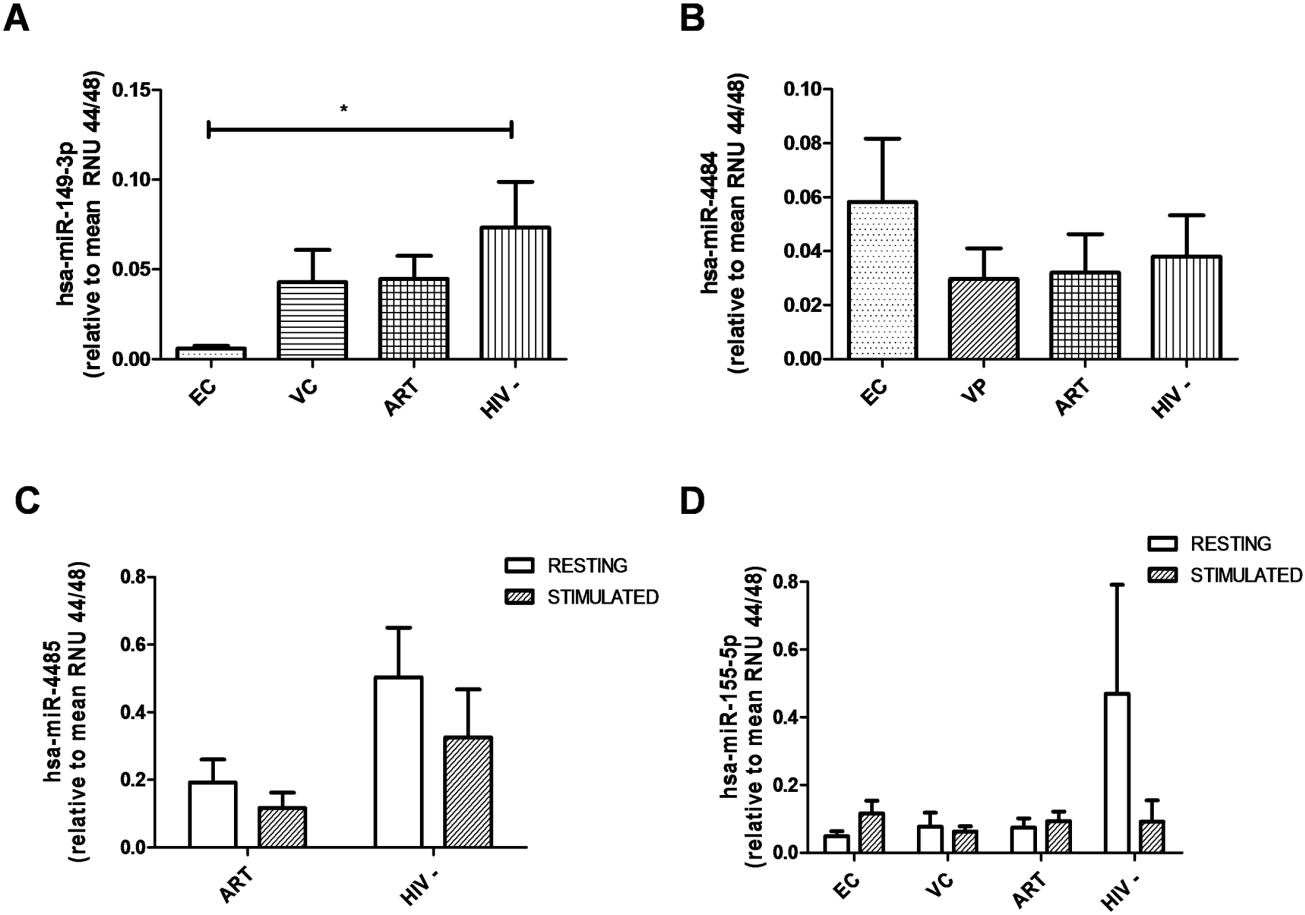
Validation analysis of the miRNA expression profiles in CD8+ T-cells through individual RT-qPCR assays, normalized to endogenous control miRNAs. Graphs represent expression levels of: A) hsa-miR-149-3p; B) hsa-miR-4484; C) hsa-miR-4485; and D) hsa-miR-155-5p. One-way analysis of variance (ANOVA) tests were performed for global comparisons and Turkey post-hoc tests for pair comparisons. Bars represent standard error means (SEM); *, p<0.05. *VP*, *viremic progressors; EC*, *elite controllers; ART*, *patients on antiretroviral therapy; HIV-*, *uninfected donors; VC*, *viremic controllers*.

Although statistically significant differences were not observed in any comparison, except for hsa-miR-149-3p between resting cells from EC and HIV- (p<0.05), overall the four miRNAs of interest reflected the same expression tendencies observed in the profiling analysis: resting CD8+ T-cells from EC, VP and ART showed downexpressed levels of hsa-miR-149-3p compared to HIV- (Fig 5A); resting CD8+ T-cells from VP and ART showed lower expression levels of hsa-miR-4484 compared to HIV- (Fig 5B); and stimulated CD8+ T-cells from ART and HIV- showed downexpressed levels of hsa-miR-4485 compared to the respective resting samples (Fig 5C). As previously mentioned, the expression levels of hsa-miR-155-5p were significantly higher in stimulated CD8+ T-cells from EC, ART, HIV- and VC than in the respective resting samples (S4 Table, Fig 3). However, the validation analysis only reproduced this expression tendency in EC (Fig 5D).

## Discussion

It is known that miRNAs are involved in CD4+ T-cell differentiation and strongly influence CD8+ T-cell responses [28,29]. However, the role of miRNAs in promoting CD8+ T-cell immunity in the field of HIV infection remains unknown. The goal of this study was to assess the differential miRNA expression pattern between CD8+ T-cells from HIV-infected patients who differ in the control of viral replication and therefore, in the progression of the disease. For that purpose, the miRNA profile of 68 individuals categorized in VP, EC, VC, ART and HIV- was obtained from resting and stimulated CD8+ T-cells.

The analysis between resting samples showed a downregulated miRNA pattern when samples from either of the HIV-infected groups were compared to the non-HIV infected group. It has been shown that the knockdown of Dicer and Drosha, the miRNA processing machinery, enhanced HIV infection of T-cell lines [30] and therefore, reduced levels of Dicer and Drosha in T-lymphocytes of HIV-infected individuals may lead to a downregulation of cellular miRNAs. Moreover, a more differentiated phenotype of CD8+ T-cells due to the continuous antigenic stimulation and the cellular immunosenescence present in HIV-infected individuals could also explain the preferential miRNA downregulation in HIV-infected patients with respect to the healthy controls [31]. The most downregulated specific miRNAs in each comparison were: hsa-miR-4734 in VP vs HIV-, hsa-miR-4505 in EC vs HIV- and hsa-miR-4492 and hsa-miR-4508 in ART vs HIV-. According to our knowledge, this is the first evidence of the association of these molecules with HIV infection. Nonetheless, some of them such as hsa-miR-4505 or hsa-miR-4492, have been recently reported to be involved in other infectious contexts [32].

A downregulated miRNA pattern was observed when stimulated samples from EC, ART and HIV- groups were compared to the VP group. Interestingly, hsa-miR-4492 was the most downregulated miRNA in all three comparisons. To date, this molecule has not been reported to be associated with HIV infection or disease progression. However, according to the miRDB database for miRNA target prediction and functional annotations [33], hsa-miR-4492 targets 2346 predicted genes including the Linker for Activation of T-Cells (LAT), a key protein in the T-Cell Receptor Signaling (TCR) pathway. The downregulation of hsa-miR-4492 after the stimulation of CD8+ T-cells might be related to the better immune response observed in EC, ART and HIV- individuals in contrast with the most vulnerable VP [34]. A previous study in stimulated PBMCs, revealed a differential miRNA profile between EC and VP [18]. However, none of the miRNAs observed in stimulated PBMCs is differentially expressed in stimulated CD8+ T-cells. Therefore, any of the remaining cell subset would be contributing to the differential pattern observed in PBMCs.

This study presented a preferential miRNA downregulation when stimulated CD8+ T-cells were compared to the respective resting samples. T-cell activation induces extensive changes in gene expression promoting the differentiation into cells that coordinate immune responses. miRNAs play a critical role in this process, and their expression also changes dramatically during T-cell differentiation. Quantitative analyses revealed that T-cell activation induces global post-transcriptional miRNA down-regulation *in vitro* and *in vivo* [35]. However, hsa-miR-155 has been reported to be upregulated in *in vitro* and *ex vivo* CD8+ T-cells after activation [36,37], in order to control cell proliferation and differentiation [38,39]. Accordingly, our results revealed an upregulation of hsa-miR-155 in every group of study unless in VP where a downexpression was observed. The lack of hsa-miR-155 results in an intrinsic defect of CD8+T cells that affects their proliferation and the responses to both virus and cancer. Reduced levels of hsa-miR-155 lead to a diminished accumulation of effector CD8+ T-cells during acute and chronic viral infections and the control of virus replication is impaired [36]. Inhibition of the type-I interferon-associated transcription factors, STAT1 or IRF7, resulted in enhanced responses of miR-155-deficient CD8+ T-cells *in vivo*. Conversely, miR-155 overexpression augmented antiviral CD8+ T-cell responses in vivo. Thus, it seems that miR-155 could play a role in regulating responsiveness to interferon and CD8+ T-cell responses to pathogens *in vivo* [40].

Similarly, hsa-miR-181a promotes CD8+ T-cell activation through targeting different phosphatases such as DUSP5, DUSP6, PTPN22, and SHP2 [41]. Indeed, the overexpression of miR-181a in mature cells not only enhances TCR sensitivity to antigens but also enables them to respond to antagonists [42]. Moreover, an under representation of hsa-miR-181a has been described to occur in aged T-lymphocytes with a subsequent impaired TCR sensitivity which supports an ineffective T-cell immune response [43]. Our results revealed a downexpression of hsa-mir-181 only in VP whereas no differences were observed in the other groups. Taken together, the miRNA profile between stimulated and resting samples from VP might exhibit a detrimental pattern in terms of CD8+ T cell immune response. It should be noted that the VPs included in this study showed a high degree of both CD4+ and CD8+ T-cell activation in terms of CD38+ expression, and a lower percentage of circulating CD8+ CD28+ T-lymphocytes concomitantly with a poor proliferative response to recall and specific antigens such as CMV and recombinant HIV p24 (data not shown).

Our study reveals that the predicted gene targets for all the differentially expressed miRNAs in each comparison, were targets involved in signal transduction, metabolic regulation, cell death and immune response pathways which are relevant for the induction of the immune response. Although not yet well known, the different metabolic pathways and metabolites might regulate lymphocyte signalling as well as T-cell differentiation and function [44]. Interestingly, most of the differential miRNAs are specifically found in VP, probably reflecting of a more deleterious effect on immune system and T-cell homeostasis due to viremia. Moreover, previous reports showed that, unlikely to what occurs for CD4+ T-cells, CD8+ T-cell transcriptional profile presents a close correlation with plasma viral load levels [45–47]. Furthermore, this is in accordance with the fact that the immune CD4+ and CD8+ T-cell responses were different at distinct stages after HIV-1 infection.

These results are of potential interest concerning the knowledge of the relationship between host miRNA, viral control and immune response in the field of HIV infection. However, some study limitations should be considered. First, this study shows data from isolated resting and mitogen-stimulated CD8+ T-cells whereas probably, analysis of HIV-specific CD8+ T-cells could help to better elucidate the miRNA profile with respect to HIV control and/or progression. Moreover, even if a complete miRNA expression profile has been analysed, a parallel mRNA transcriptome profile would have been desirable in order to complete the scenario of the different pathways involved in control or progressive status of HIV infection.

In summary, this study demonstrates the existence of a specific miRNA pattern present in stimulated CD8+ T-cells from viremic progressors which probably reflects a detrimental pattern in terms of CD8+ T cell immune response. Moreover, resting CD8+ T-cells do not exhibit a differential miRNA expression between HIV-infected individuals, independently of the viremia level, but they do differ from non-infected individuals. Further studies in other cohorts are necessary to validate the miRNA patterns and targets observed and functional studies are needed to dissect the relevant roles of the mentioned miRNAs in the scenario of HIV. Moreover, miRNA-profiling analyses in the remaining immune-cell subsets will be undertaken in order to shed more light to the miRNA patterns and roles involved in disease progression and immune response.

## Supporting Information

S1 Table Number of the differentially expressed miRNAs in every comparison. VP, viremic progressors; EC, elite controllers; ART, patients on antiretroviral therapy; HIV-, uninfected donors; VC, viremic controllers.
(DOCX)

S2 Table Differential miRNAs between resting CD8+ T-cells. VP, viremic progressors; EC, elite controllers; ART, patients on antiretroviral therapy; HIV-, uninfected donors; VC, viremic controllers; p-val, p-value; q-val, adjusted p-value; rej, rejection value.
(DOCX)

S3 Table Differential miRNAs between stimulated CD8+ T-cells. VP, viremic progressors; EC, elite controllers; ART, patients on antiretroviral therapy; HIV-, uninfected donors; VC, viremic controllers; p-val, p-value; q-val, adjusted p-value; rej, rejection value.
(DOCX)

S4 Table Differential miRNAs between resting and stimulated CD8+ T-cells. VP, viremic progressors; EC, elite controllers; ART, patients on antiretroviral therapy; HIV-, uninfected donors; VC, viremic controllers; p-val, p-value; q-val, adjusted p-value; rej, rejection value.
(DOCX)

S5 Table Number of predicted gene targets for all the differentially expressed miRNAs in each comparison. VP, viremic progressors; EC, elite controllers; ART, patients on antiretroviral therapy; HIV-, uninfected donors; VC, viremic controllers.
(DOCX)
