# Supplementary material for: MicroRNA Profile in CD8+ T-Lymphocytes from HIV-Infected Individuals: Relationship with Antiviral Immune Response and Disease Progression
Source: PLoS One. 2016 May 12;11(5):e0155245. doi: 10.1371/journal.pone.0155245 (PMC4865051; doi:10.1371/journal.pone.0155245)
Supplement: S1 Table — VP, viremic progressors; EC, elite controllers; ART, patients on antiretroviral therapy; HIV-, uninfected donors; VC, viremic controllers. (DOCX) [file pone.0155245.s001.docx]

Supplementary Table 1. Number of the differentially expressed miRNAs in every comparison.

| **resting CD8+ T-cells** | | | |
| --- | --- | --- | --- |
| **Comparison** | **Downregulated** | **Upregulated** | **Total** |
| VP vs EC | 0 | 0 | 0 |
| VP vs ART | 0 | 1 | 1 |
| VP vs HIV- | 16 | 0 | 16 |
| VP vs VC | 0 | 0 | 0 |
| EC vs ART | 0 | 0 | 0 |
| EC vs HIV- | 26 | 0 | 26 |
| EC vs VC | 0 | 0 | 0 |
| ART vs HIV- | 52 | 0 | 52 |
| ART vs VC | 0 | 0 | 0 |
| VC vs HIV- | 30 | 0 | 30 |
|  |  |  |  |
| **stimulated CD8+ T-cells** | | | |
| **Comparison** | **Downregulated** | **Upregulated** | **Total** |
| EC vs VP | 29 | 0 | 29 |
| ART vs VP | 7 | 3 | 10 |
| HIV- vs VP | 14 | 0 | 14 |
| VP vs VC | 0 | 0 | 0 |
| EC vs ART | 1 | 0 | 1 |
| EC vs HIV- | 0 | 1 | 1 |
| EC vs VC | 1 | 0 | 1 |
| ART vs HIV- | 0 | 0 | 0 |
| ART vs VC | 1 | 0 | 1 |
| HIV- vs VC | 3 | 0 | 3 |
|  |  |  |  |
| **stimulated vs resting CD8+ T-cells** | | | |
| **Comparison** | **Downregulated** | **Upregulated** | **Total** |
| stimulus vs resting VP | 14 | 3 | 17 |
| stimulus vs resting EC | 19 | 3 | 22 |
| stimulus vs resting ART | 4 | 4 | 8 |
| stimulus vs resting HIV- | 80 | 3 | 83 |
| stimulus vs resting VC | 3 | 4 | 7 |

*VP, viremic progressors; EC, elite controllers; ART, patients on antiretroviral therapy; HIV-, uninfected donors; VC, viremic controllers.*
