# Supplementary material for: MicroRNA Profile in CD8+ T-Lymphocytes from HIV-Infected Individuals: Relationship with Antiviral Immune Response and Disease Progression
Source: PLoS One. 2016 May 12;11(5):e0155245. doi: 10.1371/journal.pone.0155245 (PMC4865051; doi:10.1371/journal.pone.0155245)
Supplement: S2 Table — VP, viremic progressors; EC, elite controllers; ART, patients on antiretroviral therapy; HIV-, uninfected donors; VC, viremic controllers; p-val, p-value; q-val, adjusted p-value; rej, rejection value. (DOCX) [file pone.0155245.s002.docx]

Supplementary Table 2. Differential miRNAs between resting CD8+ T-cells.

| **resting CD8+ T-cells** | probeset | group1 | group2 | fold Change (mean) | p-val | q-val | rej |
| --- | --- | --- | --- | --- | --- | --- | --- |
| VP vs ART | MATURE_hsa-mir-4492_at | 7,683125584 | 6,959741909 | 1,651049844 | 0,032767217 | 0,992529139 | 1 |
| VP vs HIV- | MATURE_hsa-mir-4734_at | 7,015231012 | 7,826777161 | -1,755091382 | 0,010716398 | 0,591777748 | -1 |
|  | MATURE_hsa-mir-2861_at | 9,254767037 | 9,999390486 | -1,675536889 | 0,04614068 | 0,652997304 | -1 |
|  | MATURE_hsa-mir-4505_at | 7,541302975 | 8,279213605 | -1,667758773 | 0,031737141 | 0,649797624 | -1 |
|  | MATURE_hsa-mir-4651_at | 6,646955765 | 7,367549438 | -1,647859994 | 0,007979179 | 0,591777748 | -1 |
|  | MATURE_hsa-mir-3940-5p_at | 8,170629346 | 8,841758743 | -1,592319008 | 0,041474272 | 0,652997304 | -1 |
|  | HAIRPIN_hsa-mir-1469_at | 8,047772303 | 8,707676725 | -1,579977947 | 0,025446898 | 0,613643898 | -1 |
|  | MATURE_hsa-mir-1469_at | 8,047772303 | 8,707676725 | -1,579977947 | 0,025446898 | 0,613643898 | -1 |
|  | MATURE_hsa-mir-3185_at | 6,445051601 | 7,104771999 | -1,579776424 | 0,025296531 | 0,613643898 | -1 |
|  | HAIRPIN_hsa-mir-4734_at | 6,39995817 | 7,058644837 | -1,578644877 | 0,005035426 | 0,591777748 | -1 |
|  | MATURE_hsa-mir-4507_at | 7,429780518 | 8,062598343 | -1,550590601 | 0,043112515 | 0,652997304 | -1 |
|  | MATURE_hsa-mir-4674_at | 6,028327414 | 6,656430148 | -1,545531152 | 0,025925012 | 0,613643898 | -1 |
|  | HAIRPIN_hsa-mir-4507_at | 6,606921148 | 7,223482996 | -1,533216945 | 0,016707752 | 0,612801658 | -1 |
|  | MATURE_hsa-mir-1587_at | 5,92430095 | 6,53543082 | -1,527454991 | 0,008304214 | 0,591777748 | -1 |
|  | MATURE_hsa-mir-663a_at | 6,854682958 | 7,442855489 | -1,503341252 | 0,047032337 | 0,652997304 | -1 |
|  | HAIRPIN_hsa-mir-663a_at | 6,727471762 | 7,313062825 | -1,500653672 | 0,023905828 | 0,612801658 | -1 |
|  | MATURE_hsa-mir-4484_at | 5,537436523 | 6,122571973 | -1,500179829 | 0,04816613 | 0,652997304 | -1 |
| EC vs HIV- | MATURE_hsa-mir-4505_at | 7,428766986 | 8,279213605 | -1,803059017 | 0,009974562 | 0,594987648 | -1 |
|  | MATURE_hsa-mir-4530_at | 9,096756983 | 9,897360514 | -1,741829644 | 0,028916861 | 0,615205404 | -1 |
|  | MATURE_hsa-mir-4484_at | 5,339793659 | 6,122571973 | -1,720440875 | 0,006096352 | 0,594987648 | -1 |
|  | MATURE_hsa-mir-149-3p_at | 8,333294937 | 9,085951326 | -1,684892315 | 0,013373092 | 0,594987648 | -1 |
|  | HAIRPIN_hsa-mir-4530_at | 8,57823487 | 9,326472656 | -1,679739819 | 0,020437143 | 0,594987648 | -1 |
|  | MATURE_hsa-mir-4507_at | 7,329400457 | 8,062598343 | -1,662319718 | 0,014672999 | 0,594987648 | -1 |
|  | MATURE_hsa-mir-4734_at | 7,111193787 | 7,826777161 | -1,642147108 | 0,018427599 | 0,594987648 | -1 |
|  | MATURE_hsa-mir-4508_at | 9,522659307 | 10,23795316 | -1,641817591 | 0,045131694 | 0,67578356 | -1 |
|  | MATURE_hsa-mir-3620-5p_at | 7,06025449 | 7,754703557 | -1,618266335 | 0,008601175 | 0,594987648 | -1 |
|  | MATURE_hsa-mir-4492_at | 7,364409612 | 8,054608686 | -1,613506148 | 0,047356701 | 0,67578356 | -1 |
|  | HAIRPIN_hsa-mir-663a_at | 6,628389857 | 7,313062825 | -1,607337588 | 0,006020577 | 0,594987648 | -1 |
|  | MATURE_hsa-mir-663a_at | 6,764334493 | 7,442855489 | -1,600498136 | 0,017015339 | 0,594987648 | -1 |
|  | MATURE_hsa-mir-1587_at | 5,868367319 | 6,53543082 | -1,587837749 | 0,002708038 | 0,594987648 | -1 |
|  | HAIRPIN_hsa-mir-1469_at | 8,053178438 | 8,707676725 | -1,574068459 | 0,020608085 | 0,594987648 | -1 |
|  | MATURE_hsa-mir-1469_at | 8,053178438 | 8,707676725 | -1,574068459 | 0,020608085 | 0,594987648 | -1 |
|  | MATURE_hsa-mir-4763-3p_at | 7,753728216 | 8,405587398 | -1,571191665 | 0,014865226 | 0,594987648 | -1 |
|  | HAIRPIN_hsa-mir-3656_at | 8,568510558 | 9,220360198 | -1,571181274 | 0,029430993 | 0,615205404 | -1 |
|  | MATURE_hsa-mir-3656_at | 8,666383689 | 9,318096659 | -1,571032439 | 0,041622853 | 0,67578356 | -1 |
|  | HAIRPIN_hsa-mir-4507_at | 6,582469239 | 7,223482996 | -1,559424555 | 0,009446429 | 0,594987648 | -1 |
|  | MATURE_hsa-mir-4463_at | 6,807282849 | 7,441312112 | -1,551893186 | 0,015665923 | 0,594987648 | -1 |
|  | MATURE_hsa-mir-4532_at | 5,622919203 | 6,25352 | -1,548209596 | 0,01043872 | 0,594987648 | -1 |
|  | MATURE_hsa-mir-4651_at | 6,743460902 | 7,367549438 | -1,541236797 | 0,016032435 | 0,594987648 | -1 |
|  | MATURE_hsa-mir-762_at | 8,608035086 | 9,222293779 | -1,530771231 | 0,040445262 | 0,67578356 | -1 |
|  | MATURE_hsa-mir-4674_at | 6,063827534 | 6,656430148 | -1,507964655 | 0,027988332 | 0,609004081 | -1 |
|  | HAIRPIN_hsa-mir-4492_at | 7,062091583 | 7,652524511 | -1,505698514 | 0,024335174 | 0,604133519 | -1 |
|  | HAIRPIN_hsa-mir-762_at | 8,444710744 | 9,034641341 | -1,505174337 | 0,033050481 | 0,639777181 | -1 |
| ART vs HIV- | MATURE_hsa-mir-4492_at | 6,959741909 | 8,054608686 | -2,135933569 | 0,002357301 | 0,212918904 | -1 |
|  | MATURE_hsa-mir-4508_at | 9,157927362 | 10,23795316 | -2,114073881 | 0,003401822 | 0,212918904 | -1 |
|  | MATURE_hsa-mir-4505_at | 7,264938612 | 8,279213605 | -2,01988757 | 0,002794111 | 0,212918904 | -1 |
|  | MATURE_hsa-mir-663a_at | 6,48231213 | 7,442855489 | -1,946042691 | 0,001083172 | 0,189150883 | -1 |
|  | MATURE_hsa-mir-4467_at | 6,689222944 | 7,635268872 | -1,92658512 | 0,001620304 | 0,212918904 | -1 |
|  | MATURE_hsa-mir-149-3p_at | 8,185210679 | 9,085951326 | -1,867024225 | 0,003969146 | 0,212918904 | -1 |
|  | HAIRPIN_hsa-mir-663a_at | 6,420510409 | 7,313062825 | -1,856457665 | 0,00052568 | 0,189150883 | -1 |
|  | MATURE_hsa-mir-1908_at | 7,698721373 | 8,588644027 | -1,853076774 | 0,004717612 | 0,212918904 | -1 |
|  | HAIRPIN_hsa-mir-1469_at | 7,819787619 | 8,707676725 | -1,850466614 | 0,002327029 | 0,212918904 | -1 |
|  | MATURE_hsa-mir-1469_at | 7,819787619 | 8,707676725 | -1,850466614 | 0,002327029 | 0,212918904 | -1 |
|  | MATURE_hsa-mir-2861_at | 9,120923829 | 9,999390486 | -1,83842033 | 0,016611778 | 0,395955361 | -1 |
|  | MATURE_hsa-mir-4488_at | 8,674882455 | 9,544655143 | -1,827374956 | 0,016435178 | 0,394691419 | -1 |
|  | MATURE_hsa-mir-3656_at | 8,458665359 | 9,318096659 | -1,814322976 | 0,009012738 | 0,312806031 | -1 |
|  | MATURE_hsa-mir-4497_at | 8,769386279 | 9,611360095 | -1,792500866 | 0,019120458 | 0,404442015 | -1 |
|  | HAIRPIN_hsa-mir-4467_at | 6,181501584 | 7,014108961 | -1,780901071 | 0,00068559 | 0,189150883 | -1 |
|  | HAIRPIN_hsa-mir-638_at | 9,415998748 | 10,24037821 | -1,77077322 | 0,013431623 | 0,376321081 | -1 |
|  | MATURE_hsa-mir-638_at | 9,415998748 | 10,24037821 | -1,77077322 | 0,013431623 | 0,376321081 | -1 |
|  | MATURE_hsa-mir-4507_at | 7,242815815 | 8,062598343 | -1,765139895 | 0,007793855 | 0,284859797 | -1 |
|  | MATURE_hsa-mir-3620-5p_at | 6,937481239 | 7,754703557 | -1,762010251 | 0,00262202 | 0,212918904 | -1 |
|  | HAIRPIN_hsa-mir-4508_at | 5,691171617 | 6,499247281 | -1,750874483 | 0,003112416 | 0,212918904 | -1 |
|  | HAIRPIN_hsa-mir-3196_at | 9,067145333 | 9,87300109 | -1,748182447 | 0,02183983 | 0,420219382 | -1 |
|  | MATURE_hsa-mir-3196_at | 9,067145333 | 9,87300109 | -1,748182447 | 0,02183983 | 0,420219382 | -1 |
|  | MATURE_hsa-mir-4674_at | 5,857903397 | 6,656430148 | -1,739324059 | 0,004056125 | 0,212918904 | -1 |
|  | MATURE_hsa-mir-1228-5p_at | 7,358192654 | 8,153945358 | -1,735982861 | 0,004558018 | 0,212918904 | -1 |
|  | MATURE_hsa-mir-3940-5p_at | 8,052905879 | 8,841758743 | -1,727700163 | 0,01470167 | 0,38428841 | -1 |
|  | HAIRPIN_hsa-mir-3656_at | 8,437831946 | 9,220360198 | -1,720142698 | 0,010892183 | 0,337763429 | -1 |
|  | HAIRPIN_hsa-mir-1908_at | 7,52504439 | 8,291667183 | -1,701282583 | 0,002683121 | 0,212918904 | -1 |
|  | HAIRPIN_hsa-mir-4492_at | 6,88975445 | 7,652524511 | -1,696745352 | 0,004708526 | 0,212918904 | -1 |
|  | MATURE_hsa-mir-4530_at | 9,137055014 | 9,897360514 | -1,69384927 | 0,042285488 | 0,521466592 | -1 |
|  | MATURE_hsa-mir-1587_at | 5,787189775 | 6,53543082 | -1,679743614 | 0,001051563 | 0,189150883 | -1 |
|  | HAIRPIN_hsa-mir-4507_at | 6,489160194 | 7,223482996 | -1,663616388 | 0,003775522 | 0,212918904 | -1 |
|  | HAIRPIN_hsa-mir-4497_at | 6,995294455 | 7,72481006 | -1,658082286 | 0,00784836 | 0,284859797 | -1 |
|  | MATURE_hsa-mir-4734_at | 7,103968072 | 7,826777161 | -1,650392406 | 0,019923318 | 0,415191893 | -1 |
|  | MATURE_hsa-mir-4787-5p_at | 10,74672677 | 11,4689599 | -1,649733664 | 0,02836729 | 0,460903988 | -1 |
|  | MATURE_hsa-mir-762_at | 8,509050695 | 9,222293779 | -1,639485434 | 0,020335034 | 0,418190188 | -1 |
|  | MATURE_hsa-mir-4466_at | 9,703595426 | 10,41660846 | -1,639224021 | 0,045599772 | 0,542300666 | -1 |
|  | HAIRPIN_hsa-mir-4466_at | 8,836390376 | 9,520852428 | -1,607102617 | 0,026704855 | 0,459646658 | -1 |
|  | MATURE_hsa-mir-3621_at | 4,969998251 | 5,652034596 | -1,604402751 | 0,000882834 | 0,189150883 | -1 |
|  | HAIRPIN_hsa-mir-4530_at | 8,645660399 | 9,326472656 | -1,603042035 | 0,038773034 | 0,511891311 | -1 |
|  | MATURE_hsa-mir-371b-5p_at | 4,483044048 | 5,162838331 | -1,601911319 | 0,003773943 | 0,212918904 | -1 |
|  | MATURE_hsa-mir-4463_at | 6,764619471 | 7,441312112 | -1,598471078 | 0,011784775 | 0,354716342 | -1 |
|  | HAIRPIN_hsa-mir-762_at | 8,361653005 | 9,034641341 | -1,594372063 | 0,017716374 | 0,399343793 | -1 |
|  | MATURE_hsa-mir-4651_at | 6,72266143 | 7,367549438 | -1,563617902 | 0,015022094 | 0,384420222 | -1 |
|  | MATURE_hsa-mir-4745-5p_at | 7,143347019 | 7,778724666 | -1,553344308 | 0,045671302 | 0,542300666 | -1 |
|  | MATURE_hsa-mir-4532_at | 5,632481736 | 6,25352 | -1,537981622 | 0,01359628 | 0,377621903 | -1 |
|  | MATURE_hsa-mir-4484_at | 5,507536932 | 6,122571973 | -1,531595196 | 0,034032625 | 0,502281562 | -1 |
|  | MATURE_hsa-mir-3185_at | 6,491404053 | 7,104771999 | -1,529826396 | 0,033397176 | 0,502281562 | -1 |
|  | HAIRPIN_hsa-mir-4505_at | 4,993870444 | 5,604360298 | -1,526777525 | 0,002902454 | 0,212918904 | -1 |
|  | MATURE_hsa-mir-4758-5p_at | 5,783654443 | 6,393781029 | -1,526393134 | 0,002697625 | 0,212918904 | -1 |
|  | HAIRPIN_hsa-mir-4674_at | 5,662692976 | 6,27152309 | -1,525022062 | 0,003603211 | 0,212918904 | -1 |
|  | MATURE_hsa-mir-4707-5p_at | 6,651160272 | 7,256011125 | -1,520821521 | 0,017065824 | 0,399343793 | -1 |
|  | MATURE_hsa-mir-1202_at | 5,109639092 | 5,710417655 | -1,516534757 | 0,006971384 | 0,269787011 | -1 |
| VC vs HIV- | MATURE_hsa-mir-4505_at | 8,279213605 | 7,272939304 | -2,008716969 | 0,003097483 | 0,21507307 | 1 |
|  | MATURE_hsa-mir-4516_at | 9,567040184 | 8,672608661 | -1,858877273 | 0,011712657 | 0,366766935 | 1 |
|  | MATURE_hsa-mir-4530_at | 9,897360514 | 9,009836868 | -1,849997917 | 0,018467351 | 0,416133419 | 1 |
|  | MATURE_hsa-mir-4734_at | 7,826777161 | 6,998366172 | -1,775728464 | 0,00803277 | 0,321277441 | 1 |
|  | MATURE_hsa-mir-149-3p_at | 9,085951326 | 8,261503899 | -1,770856644 | 0,008392718 | 0,32687067 | 1 |
|  | HAIRPIN_hsa-mir-4530_at | 9,326472656 | 8,50214848 | -1,770705364 | 0,012970462 | 0,383589391 | 1 |
|  | MATURE_hsa-mir-4651_at | 7,367549438 | 6,54988937 | -1,76254497 | 0,002273055 | 0,190380465 | 1 |
|  | MATURE_hsa-mir-4507_at | 8,062598343 | 7,264508308 | -1,738797631 | 0,009749977 | 0,349681362 | 1 |
|  | HAIRPIN_hsa-mir-4507_at | 7,223482996 | 6,464426863 | -1,69238304 | 0,002868747 | 0,211752673 | 1 |
|  | MATURE_hsa-mir-4758-5p_at | 6,393781029 | 5,644529528 | -1,680920509 | 0,00027231 | 0,08767326 | 1 |
|  | MATURE_hsa-mir-3620-5p_at | 7,754703557 | 7,028998377 | -1,653708761 | 0,007489653 | 0,321277441 | 1 |
|  | HAIRPIN_hsa-mir-4734_at | 7,058644837 | 6,359131631 | -1,623956745 | 0,002477282 | 0,19298629 | 1 |
|  | MATURE_hsa-mir-1972_at | 5,396764077 | 4,698176023 | -1,622915689 | 0,008047024 | 0,321277441 | 1 |
|  | MATURE_hsa-mir-1587_at | 6,53543082 | 5,83971478 | -1,619688119 | 0,002317727 | 0,190380465 | 1 |
|  | MATURE_hsa-mir-4463_at | 7,441312112 | 6,755851462 | -1,6082154 | 0,011003549 | 0,361397759 | 1 |
|  | HAIRPIN_hsa-mir-1469_at | 8,707676725 | 8,024731494 | -1,60541383 | 0,018615983 | 0,416133419 | 1 |
|  | MATURE_hsa-mir-1469_at | 8,707676725 | 8,024731494 | -1,60541383 | 0,018615983 | 0,416133419 | 1 |
|  | MATURE_hsa-mir-1910_at | 5,731963399 | 5,058440215 | -1,594963251 | 0,000205572 | 0,08767326 | 1 |
|  | MATURE_hsa-mir-4763-3p_at | 8,405587398 | 7,733088155 | -1,59383164 | 0,014354586 | 0,393439688 | 1 |
|  | MATURE_hsa-mir-762_at | 9,222293779 | 8,566614605 | -1,575357406 | 0,033173214 | 0,488609259 | 1 |
|  | MATURE_hsa-mir-1915-5p_at | 4,321913837 | 3,669406594 | -1,571897606 | 1,63E-05 | 0,05210703 | 1 |
|  | MATURE_hsa-mir-4741_at | 6,483288675 | 5,837131183 | -1,564994397 | 0,009895896 | 0,349681362 | 1 |
|  | HAIRPIN_hsa-mir-3656_at | 9,220360198 | 8,580986388 | -1,557652926 | 0,03723588 | 0,498072786 | 1 |
|  | MATURE_hsa-mir-663a_at | 7,442855489 | 6,813094777 | -1,547308334 | 0,030550298 | 0,487083208 | 1 |
|  | HAIRPIN_hsa-mir-4516_at | 6,671161702 | 6,04339477 | -1,545171456 | 0,005412251 | 0,298047056 | 1 |
|  | MATURE_hsa-mir-1228-5p_at | 8,153945358 | 7,538187604 | -1,532362636 | 0,027603548 | 0,476571518 | 1 |
|  | HAIRPIN_hsa-mir-663a_at | 7,313062825 | 6,697778369 | -1,531860005 | 0,015783652 | 0,406556329 | 1 |
|  | HAIRPIN_hsa-mir-762_at | 9,034641341 | 8,419654618 | -1,531543903 | 0,030418403 | 0,487083208 | 1 |
|  | MATURE_hsa-mir-4674_at | 6,656430148 | 6,044339234 | -1,528472837 | 0,027004498 | 0,474911466 | 1 |
|  | HAIRPIN_hsa-mir-4505_at | 5,604360298 | 5,002878661 | -1,517273996 | 0,003426012 | 0,23282303 | 1 |

*VP, viremic progressors; EC, elite controllers; ART, patients on antiretroviral therapy; HIV-, uninfected donors; VC, viremic controllers; p-val, p-value; q-val, adjusted p-value; rej, rejection value.*
