# Supplementary material for: MicroRNA Profile in CD8+ T-Lymphocytes from HIV-Infected Individuals: Relationship with Antiviral Immune Response and Disease Progression
Source: PLoS One. 2016 May 12;11(5):e0155245. doi: 10.1371/journal.pone.0155245 (PMC4865051; doi:10.1371/journal.pone.0155245)
Supplement: S3 Table — VP, viremic progressors; EC, elite controllers; ART, patients on antiretroviral therapy; HIV-, uninfected donors; VC, viremic controllers; p-val, p-value; q-val, adjusted p-value; rej, rejection value. (DOCX) [file pone.0155245.s003.docx]

Supplementary Table 3. Differential miRNAs between stimulated CD8+ T-cells.

| **stimulated CD8+ T-cells** | probeset | group1 | group2 | fold Change (mean) | p-val | q-val | rej |
| --- | --- | --- | --- | --- | --- | --- | --- |
| EC vs VP | MATURE_hsa-mir-663b_at | 4,833338357 | 4,243221488 | -1,505368688 | 5,37E-05 | 0,171546685 | 1 |
|  | MATURE_hsa-mir-3687_at | 5,1635314 | 4,566311613 | -1,512798447 | 0,001103408 | 0,446738059 | 1 |
|  | MATURE_hsa-mir-1908_at | 8,051134379 | 7,448641169 | -1,518338233 | 0,040281014 | 0,677203189 | 1 |
|  | MATURE_hsa-mir-92b-5p_at | 5,631962912 | 5,014649442 | -1,534015935 | 0,001183026 | 0,446738059 | 1 |
|  | HAIRPIN_hsa-mir-638_at | 9,461674415 | 8,838863263 | -1,539872766 | 0,046009108 | 0,677203189 | 1 |
|  | MATURE_hsa-mir-638_at | 9,461674415 | 8,838863263 | -1,539872766 | 0,046009108 | 0,677203189 | 1 |
|  | HAIRPIN_hsa-mir-4497_at | 7,658830653 | 7,029137702 | -1,54723566 | 0,014434572 | 0,635937917 | 1 |
|  | HAIRPIN_hsa-mir-3960_at | 11,36627409 | 10,71428002 | -1,571338572 | 0,038615701 | 0,677203189 | 1 |
|  | HAIRPIN_hsa-mir-4466_at | 9,018090606 | 8,360968185 | -1,576934154 | 0,023717768 | 0,65772864 | 1 |
|  | MATURE_hsa-mir-4787-5p_at | 10,89139996 | 10,23324134 | -1,578067177 | 0,033499771 | 0,677203189 | 1 |
|  | MATURE_hsa-mir-3960_at | 11,41939262 | 10,74918017 | -1,591307285 | 0,039319988 | 0,677203189 | 1 |
|  | HAIRPIN_hsa-mir-3196_at | 9,119389532 | 8,446854622 | -1,593871045 | 0,041333718 | 0,677203189 | 1 |
|  | MATURE_hsa-mir-3196_at | 9,119389532 | 8,446854622 | -1,593871045 | 0,041333718 | 0,677203189 | 1 |
|  | MATURE_hsa-mir-4505_at | 7,829123308 | 7,135722968 | -1,617090408 | 0,028335908 | 0,675409637 | 1 |
|  | MATURE_hsa-mir-4484_at | 7,238235106 | 6,530362023 | -1,633394283 | 0,009791639 | 0,577945117 | 1 |
|  | MATURE_hsa-mir-3665_at | 11,3160075 | 10,59341726 | -1,650142063 | 0,042000663 | 0,677203189 | 1 |
|  | MATURE_hsa-mir-4507_at | 7,951347611 | 7,22600509 | -1,65329311 | 0,012133257 | 0,634989965 | 1 |
|  | HAIRPIN_hsa-mir-4508_at | 6,533437231 | 5,803313424 | -1,658781437 | 0,004441083 | 0,507849251 | 1 |
|  | MATURE_hsa-mir-4466_at | 9,799606674 | 9,068028368 | -1,660454632 | 0,029388848 | 0,677203189 | 1 |
|  | MATURE_hsa-mir-2861_at | 9,155094858 | 8,413151169 | -1,672427521 | 0,031042711 | 0,677203189 | 1 |
|  | MATURE_hsa-mir-3620-5p_at | 7,591570476 | 6,845568898 | -1,677138204 | 0,003450379 | 0,507849251 | 1 |
|  | MATURE_hsa-mir-1915-3p_at | 9,833304824 | 9,068751861 | -1,698843509 | 0,029878908 | 0,677203189 | 1 |
|  | MATURE_hsa-mir-149-3p_at | 8,693558848 | 7,886103781 | -1,750121481 | 0,005945762 | 0,530874484 | 1 |
|  | HAIRPIN_hsa-mir-4492_at | 7,759025197 | 6,94457793 | -1,758624248 | 0,001398679 | 0,446738059 | 1 |
|  | MATURE_hsa-mir-4497_at | 9,681674083 | 8,841579194 | -1,790167882 | 0,01302131 | 0,635937917 | 1 |
|  | HAIRPIN_hsa-mir-4530_at | 8,744273868 | 7,882774649 | -1,816925442 | 0,005744356 | 0,530874484 | 1 |
|  | MATURE_hsa-mir-4530_at | 9,300154737 | 8,301805743 | -1,997712529 | 0,004896095 | 0,507849251 | 1 |
|  | MATURE_hsa-mir-4508_at | 10,29013817 | 9,282345412 | -2,010832288 | 0,003641847 | 0,507849251 | 1 |
|  | MATURE_hsa-mir-4492_at | 8,347625626 | 7,294646772 | 2,074809461 | 0,001882807 | 0,462591106 | 1 |
| ART vs VP | MATURE_hsa-mir-4485_at | 5,29154883 | 6,08935868 | 1,738459972 | 0,002176188 | 0,389016853 | -1 |
|  | MATURE_hsa-mir-320b_at | 6,105724174 | 6,708683911 | 1,518829302 | 0,024809729 | 0,466113171 | -1 |
|  | MATURE_hsa-mir-320a_at | 6,119939837 | 6,720538162 | 1,516345306 | 0,026033733 | 0,466113171 | -1 |
|  | MATURE_hsa-mir-4467_at | 6,958848562 | 6,371467852 | -1,50251637 | 0,037205439 | 0,50954167 | 1 |
|  | MATURE_hsa-mir-4507_at | 7,951347611 | 7,346678902 | -1,520629526 | 0,037799268 | 0,510022244 | 1 |
|  | MATURE_hsa-mir-4484_at | 7,238235106 | 6,629512104 | -1,524908843 | 0,027403566 | 0,466113171 | 1 |
|  | HAIRPIN_hsa-mir-4508_at | 6,533437231 | 5,920188037 | -1,529700478 | 0,017500075 | 0,466113171 | 1 |
|  | MATURE_hsa-mir-3620-5p_at | 7,591570476 | 6,945664742 | -1,56472132 | 0,011852127 | 0,466113171 | 1 |
|  | MATURE_hsa-mir-4508_at | 10,29013817 | 9,506657265 | -1,721278936 | 0,024321657 | 0,466113171 | 1 |
|  | MATURE_hsa-mir-4492_at | 8,347625626 | 7,320469191 | -2,038003367 | 0,002670229 | 0,397784885 | 1 |
| HIV- vs VP | MATURE_hsa-mir-92b-5p_at | 5,631962912 | 5,026769181 | -1,521183009 | 0,003316658 | 0,484562563 | 1 |
|  | MATURE_hsa-mir-3178_at | 6,657734581 | 6,035422557 | -1,53934011 | 0,012924615 | 0,68067317 | 1 |
|  | HAIRPIN_hsa-mir-4466_at | 9,018090606 | 8,369512587 | -1,56762232 | 0,039628747 | 0,702773256 | 1 |
|  | MATURE_hsa-mir-4505_at | 7,829123308 | 7,141630316 | -1,610482509 | 0,045128209 | 0,707006068 | 1 |
|  | MATURE_hsa-mir-3620-5p_at | 7,591570476 | 6,894242468 | -1,621498858 | 0,011518216 | 0,68067317 | 1 |
|  | MATURE_hsa-mir-149-3p_at | 8,693558848 | 7,989528091 | -1,629049856 | 0,026493456 | 0,68067317 | 1 |
|  | HAIRPIN_hsa-mir-3196_at | 9,119389532 | 8,41360314 | -1,631033475 | 0,048720459 | 0,707426439 | 1 |
|  | MATURE_hsa-mir-3196_at | 9,119389532 | 8,41360314 | -1,631033475 | 0,048720459 | 0,707426439 | 1 |
|  | MATURE_hsa-mir-4507_at | 7,951347611 | 7,223032625 | -1,656702995 | 0,020256383 | 0,68067317 | 1 |
|  | HAIRPIN_hsa-mir-4492_at | 7,759025197 | 7,014600259 | -1,675306354 | 0,006868241 | 0,614545707 | 1 |
|  | MATURE_hsa-mir-4530_at | 9,300154737 | 8,539134613 | -1,694688509 | 0,046613989 | 0,707426439 | 1 |
|  | HAIRPIN_hsa-mir-4508_at | 6,533437231 | 5,753235728 | -1,717370724 | 0,005131595 | 0,585368408 | 1 |
|  | MATURE_hsa-mir-4508_at | 10,29013817 | 9,338844053 | -1,933606354 | 0,01121514 | 0,68067317 | 1 |
|  | MATURE_hsa-mir-4492_at | 8,347625626 | 7,2746111 | -2,10382474 | 0,003489336 | 0,484562563 | 1 |
| EC vs ART | MATURE_hsa-mir-4485_at | 5,500679701 | 6,08935868 | -1,503869081 | 0,01750366 | 0,826325776 | -1 |
| EC vs HIV- | MATURE_hsa-mir-3135b_at | 6,459693286 | 5,749902521 | 1,635566893 | 0,031524115 | 0,997167449 | 1 |
| EC vs VC | MATURE_hsa-mir-4508_at | 9,282345412 | 9,927307665 | -1,563698373 | 0,053065444 | 0,682090111 | 0 |
| ART vs VC | MATURE_hsa-mir-4298_at | 6,368389954 | 7,049144615 | -1,602978039 | 0,0114139 | 0,999121358 | -1 |
| HIV- vs VC | MATURE_hsa-mir-3135b_at | 5,749902521 | 6,567697812 | -1,762710181 | 0,016022139 | 0,924381463 | -1 |
|  | MATURE_hsa-mir-4298_at | 6,381370539 | 7,049144615 | -1,588620005 | 0,021594271 | 0,924381463 | -1 |
|  | MATURE_hsa-mir-4739_at | 6,550622822 | 7,16088064 | -1,526531986 | 0,012134419 | 0,924381463 | -1 |

*VP, viremic progressors; EC, elite controllers; ART, patients on antiretroviral therapy; HIV-, uninfected donors; VC, viremic controllers; p-val, p-value; q-val, adjusted p-value; rej, rejection value.*
