# Supplementary material for: MicroRNA Profile in CD8+ T-Lymphocytes from HIV-Infected Individuals: Relationship with Antiviral Immune Response and Disease Progression
Source: PLoS One. 2016 May 12;11(5):e0155245. doi: 10.1371/journal.pone.0155245 (PMC4865051; doi:10.1371/journal.pone.0155245)
Supplement: S4 Table — VP, viremic progressors; EC, elite controllers; ART, patients on antiretroviral therapy; HIV-, uninfected donors; VC, viremic controllers; p-val, p-value; q-val, adjusted p-value; rej, rejection value. (DOCX) [file pone.0155245.s004.docx]

Supplementary Table 4. Differential miRNAs between resting and stimulated CD8+ T-cells.

| **stimulated vs resting** |  |  |  |  |  |  |  |
| --- | --- | --- | --- | --- | --- | --- | --- |
| **CD8+ T-cells** | probeset | group1 | group2 | fold Change (mean) | p-val | q-val | rej |
| VP | MATURE_hsa-mir-181a-5p_at | 4,927819052 | 5,837737429 | -1,878939191 | 0,004605555 | 0,341965327 | 0 |
|  | MATURE_hsa-let-7b-5p_at | 5,420574204 | 6,307337812 | -1,849023561 | 0,001167224 | 0,242740491 | 0 |
|  | HAIRPIN_hsa-mir-181a-2_at | 4,961371561 | 5,807620193 | -1,797820061 | 0,00546031 | 0,341965327 | 0 |
|  | MATURE_hsa-mir-23a-5p_at | 4,130046668 | 4,953402338 | -1,769517059 | 0,005179182 | 0,341965327 | 0 |
|  | MATURE_hsa-mir-150-5p_at | 5,620841178 | 6,428170168 | -1,749968545 | 0,002815795 | 0,275727671 | 0 |
|  | MATURE_hsa-mir-3609_at | 5,934232542 | 6,68005585 | -1,676930976 | 0,005096197 | 0,341965327 | 0 |
|  | HAIRPIN_hsa-mir-150_at | 5,545894279 | 6,255154353 | -1,634965364 | 0,002809893 | 0,275727671 | 0 |
|  | MATURE_hsa-mir-342-3p_at | 5,061921081 | 5,738557014 | -1,598408248 | 0,020155496 | 0,493308704 | 0 |
|  | HAIRPIN_hsa-let-7b_at | 5,442281621 | 6,117397661 | -1,596725197 | 0,001735445 | 0,275727671 | 0 |
|  | MATURE_hsa-mir-423-3p_at | 4,905757398 | 5,549421144 | -1,56229159 | 0,000596578 | 0,242740491 | 0 |
|  | HAIRPIN_hsa-mir-3609_at | 5,925270178 | 6,559360605 | -1,551958982 | 0,001344271 | 0,242740491 | 0 |
|  | MATURE_hsa-mir-24-3p_at | 4,390425205 | 5,024266128 | -1,551690604 | 0,000314973 | 0,222081183 | 0 |
|  | HAIRPIN_hsa-mir-181a-1_at | 4,899083097 | 5,518637526 | -1,536400596 | 0,008567652 | 0,42100126 | 0 |
|  | HAIRPIN_hsa-mir-342_at | 5,041126654 | 5,63614769 | -1,51049461 | 0,007071505 | 0,371490432 | 0 |
|  | HAIRPIN_hsa-mir-4484_at | 4,536050159 | 3,826539759 | 1,635249077 | 4,29E-07 | 0,00068535 | 1 |
|  | MATURE_hsa-mir-4497_at | 9,239595267 | 8,491420723 | 1,679666187 | 0,014483763 | 0,447291826 | 0 |
|  | MATURE_hsa-mir-4484_at | 6,852515137 | 5,151716554 | 3,250808526 | 2,26E-08 | 7,23E-05 | 1 |
| EC | MATURE_hsa-mir-3665_at | 10,29031538 | 11,23479636 | -1,924496411 | 0,029956205 | 0,433359454 | 0 |
|  | MATURE_hsa-mir-2861_at | 7,993391295 | 8,924217554 | -1,906367497 | 0,004911407 | 0,298602959 | 0 |
|  | HAIRPIN_hsa-mir-638_at | 8,488592441 | 9,359951152 | -1,82938498 | 0,003041558 | 0,255650967 | 0 |
|  | MATURE_hsa-mir-638_at | 8,488592441 | 9,359951152 | -1,82938498 | 0,003041558 | 0,255650967 | 0 |
|  | MATURE_hsa-mir-1202_at | 4,058429912 | 4,913889952 | -1,809335616 | 1,17E-05 | 0,009323274 | -1 |
|  | MATURE_hsa-mir-1915-3p_at | 8,707076322 | 9,553088361 | -1,797525254 | 0,015609043 | 0,40106825 | 0 |
|  | HAIRPIN_hsa-mir-3196_at | 8,083270663 | 8,919446127 | -1,785311064 | 0,01350256 | 0,380944751 | 0 |
|  | MATURE_hsa-mir-3196_at | 8,083270663 | 8,919446127 | -1,785311064 | 0,01350256 | 0,380944751 | 0 |
|  | MATURE_hsa-mir-4787-5p_at | 9,899663691 | 10,72359786 | -1,770226755 | 0,006260159 | 0,299615208 | 0 |
|  | MATURE_hsa-mir-4467_at | 6,048751076 | 6,824738211 | -1,7123613 | 0,000539672 | 0,143642703 | 0 |
|  | HAIRPIN_hsa-mir-3960_at | 10,4750861 | 11,23384956 | -1,692039747 | 0,047160497 | 0,466562817 | 0 |
|  | MATURE_hsa-mir-4466_at | 8,711874153 | 9,456784928 | -1,67587062 | 0,033925591 | 0,433359454 | 0 |
|  | HAIRPIN_hsa-mir-1202_at | 4,080933899 | 4,785086977 | -1,629187983 | 1,03E-05 | 0,009323274 | -1 |
|  | MATURE_hsa-mir-3940-5p_at | 7,225726086 | 7,880562037 | -1,574436914 | 0,016078624 | 0,404759242 | 0 |
|  | HAIRPIN_hsa-mir-4467_at | 5,669220724 | 6,309301248 | -1,558416141 | 0,000498881 | 0,143642703 | 0 |
|  | MATURE_hsa-mir-4687-3p_at | 7,964367877 | 8,604344977 | -1,558304424 | 0,017643594 | 0,415509996 | 0 |
|  | HAIRPIN_hsa-mir-4466_at | 8,030929022 | 8,667737832 | -1,554886 | 0,023207109 | 0,433359454 | 0 |
|  | MATURE_hsa-mir-1908_at | 7,104448678 | 7,732423375 | -1,545393994 | 0,009711874 | 0,326283185 | 0 |
|  | MATURE_hsa-mir-371b-5p_at | 4,190832682 | 4,79454663 | -1,519623521 | 0,00121428 | 0,200237007 | 0 |
|  | HAIRPIN_hsa-mir-155_at | 5,03860045 | 3,87891112 | 2,234093135 | 0,001390623 | 0,200237007 | 0 |
|  | MATURE_hsa-mir-4484_at | 6,143733088 | 4,953164725 | 2,282426438 | 3,11E-06 | 0,004967665 | 1 |
|  | MATURE_hsa-mir-155-5p_at | 5,113980513 | 3,882894297 | 2,347436635 | 0,001032734 | 0,200237007 | 0 |
| ART | MATURE_hsa-mir-3609_at | 5,565169599 | 6,338771536 | -1,709532603 | 0,01472496 | 0,574546229 | 0 |
|  | MATURE_hsa-mir-342-3p_at | 4,992298716 | 5,75836914 | -1,700631333 | 0,006658137 | 0,512769859 | 0 |
|  | MATURE_hsa-mir-23a-5p_at | 4,190161606 | 4,935969174 | -1,676912682 | 0,006029588 | 0,512769859 | 0 |
|  | HAIRPIN_hsa-mir-3609_at | 5,624033255 | 6,286700385 | -1,583006447 | 0,019564632 | 0,574546229 | 0 |
|  | MATURE_hsa-mir-4485_at | 5,499447631 | 4,802384854 | 1,621200783 | 0,003369528 | 0,479316532 | 0 |
|  | HAIRPIN_hsa-mir-155_at | 5,007903738 | 4,288208366 | 1,646834266 | 0,048308491 | 0,652570589 | 0 |
|  | MATURE_hsa-mir-155-5p_at | 5,104685832 | 4,333583022 | 1,706573806 | 0,040284271 | 0,646623943 | 0 |
|  | MATURE_hsa-mir-4484_at | 6,219876587 | 5,097901415 | 2,176447427 | 0,001745105 | 0,426610561 | 0 |
| HIV- | HAIRPIN_hsa-mir-3196_at | 8,067123402 | 9,526521352 | -2,749935824 | 0,000114375 | 0,038251126 | -1 |
|  | MATURE_hsa-mir-3196_at | 8,067123402 | 9,526521352 | -2,749935824 | 0,000114375 | 0,038251126 | -1 |
|  | MATURE_hsa-mir-2861_at | 8,121641997 | 9,558625075 | -2,707540799 | 0,000217016 | 0,038251126 | -1 |
|  | MATURE_hsa-mir-4530_at | 8,04303311 | 9,401259011 | -2,563697247 | 0,000145275 | 0,038251126 | -1 |
|  | MATURE_hsa-mir-1915-3p_at | 8,783252043 | 10,12347855 | -2,531910672 | 0,000237753 | 0,038251126 | -1 |
|  | HAIRPIN_hsa-mir-638_at | 8,568566233 | 9,868721246 | -2,462553407 | 0,000367476 | 0,038251126 | -1 |
|  | MATURE_hsa-mir-638_at | 8,568566233 | 9,868721246 | -2,462553407 | 0,000367476 | 0,038251126 | -1 |
|  | MATURE_hsa-mir-4466_at | 8,764990229 | 10,05607913 | -2,447126875 | 0,000348715 | 0,038251126 | -1 |
|  | MATURE_hsa-mir-1202_at | 4,163632504 | 5,447133785 | -2,434290393 | 0,000124581 | 0,038251126 | -1 |
|  | MATURE_hsa-mir-3940-5p_at | 7,225477798 | 8,479989562 | -2,385863927 | 0,00039084 | 0,038810814 | -1 |
|  | MATURE_hsa-mir-4734_at | 6,143715617 | 7,397861738 | -2,38525932 | 0,00134973 | 0,068429145 | 0 |
|  | MATURE_hsa-mir-4516_at | 8,035167159 | 9,283084989 | -2,374984059 | 0,000927439 | 0,05922877 | 0 |
|  | MATURE_hsa-mir-4787-5p_at | 9,920560395 | 11,13525105 | -2,320910116 | 0,000214037 | 0,038251126 | -1 |
|  | MATURE_hsa-mir-3656_at | 7,739090134 | 8,9351466 | -2,291125469 | 0,000432212 | 0,039447154 | -1 |
|  | MATURE_hsa-mir-3665_at | 10,4741664 | 11,66019874 | -2,275261464 | 9,13E-05 | 0,038251126 | -1 |
|  | HAIRPIN_hsa-mir-3656_at | 7,674714758 | 8,851005734 | -2,259950191 | 0,000349029 | 0,038251126 | -1 |
|  | MATURE_hsa-mir-4488_at | 8,041833633 | 9,204373785 | -2,238512155 | 0,001107257 | 0,060877422 | 0 |
|  | HAIRPIN_hsa-mir-4530_at | 7,694098552 | 8,848997941 | -2,226687946 | 0,00034122 | 0,038251126 | -1 |
|  | HAIRPIN_hsa-mir-4466_at | 8,035636433 | 9,186976274 | -2,221200831 | 0,000621997 | 0,052280444 | 0 |
|  | MATURE_hsa-mir-4505_at | 6,528030306 | 7,665613595 | -2,20012164 | 0,003453981 | 0,107686749 | 0 |
|  | MATURE_hsa-mir-3960_at | 10,54665286 | 11,65515167 | -2,156211662 | 0,000158109 | 0,038251126 | -1 |
|  | MATURE_hsa-mir-4467_at | 6,160256649 | 7,262192556 | -2,146425215 | 0,00365011 | 0,108957489 | 0 |
|  | MATURE_hsa-mir-149-3p_at | 7,558742162 | 8,655165397 | -2,138239173 | 0,000371254 | 0,038251126 | -1 |
|  | HAIRPIN_hsa-mir-1202_at | 4,215729013 | 5,311700437 | -2,137569641 | 0,000149566 | 0,038251126 | -1 |
|  | HAIRPIN_hsa-mir-3960_at | 10,52197666 | 11,61401952 | -2,131756796 | 0,000133876 | 0,038251126 | -1 |
|  | MATURE_hsa-mir-1908_at | 7,152922359 | 8,241374031 | -2,126456984 | 0,00283375 | 0,099225386 | 0 |
|  | MATURE_hsa-mir-4687-3p_at | 7,961523737 | 9,045911125 | -2,120474874 | 0,000124255 | 0,038251126 | -1 |
|  | HAIRPIN_hsa-mir-1469_at | 7,378820981 | 8,452226149 | -2,104394475 | 0,00103527 | 0,059937044 | 0 |
|  | MATURE_hsa-mir-1469_at | 7,378820981 | 8,452226149 | -2,104394475 | 0,00103527 | 0,059937044 | 0 |
|  | MATURE_hsa-mir-663a_at | 6,128187907 | 7,193978109 | -2,09331613 | 0,001868192 | 0,079551804 | 0 |
|  | MATURE_hsa-mir-4763-3p_at | 7,03782221 | 8,09005237 | -2,073733008 | 0,000644395 | 0,0527743 | 0 |
|  | MATURE_hsa-mir-3185_at | 5,72572086 | 6,769650644 | -2,061836285 | 0,002484171 | 0,091800205 | 0 |
|  | MATURE_hsa-mir-1228-5p_at | 6,812653116 | 7,844325472 | -2,044392715 | 0,001598358 | 0,071903614 | 0 |
|  | HAIRPIN_hsa-mir-663a_at | 6,055799286 | 7,063812508 | -2,011139592 | 0,001491215 | 0,070043269 | 0 |
|  | MATURE_hsa-mir-4651_at | 6,005243514 | 6,992552593 | -1,982483803 | 0,004695802 | 0,124994824 | 0 |
|  | HAIRPIN_hsa-mir-1915_at | 4,909109966 | 5,895686804 | -1,981477847 | 0,000400988 | 0,038810814 | -1 |
|  | HAIRPIN_hsa-mir-1908_at | 7,02403234 | 7,993029687 | -1,957479702 | 0,002461254 | 0,091800205 | 0 |
|  | HAIRPIN_hsa-mir-4734_at | 5,764481514 | 6,728198394 | -1,950328145 | 0,001038869 | 0,059937044 | 0 |
|  | HAIRPIN_hsa-mir-4467_at | 5,759416503 | 6,720667113 | -1,946996931 | 0,003472678 | 0,107686749 | 0 |
|  | MATURE_hsa-mir-4707-5p_at | 6,108641602 | 7,059678709 | -1,933261918 | 0,001287349 | 0,067406442 | 0 |
|  | MATURE_hsa-mir-4741_at | 5,217009127 | 6,163506552 | -1,927188146 | 0,00073741 | 0,054342095 | 0 |
|  | MATURE_hsa-mir-4674_at | 5,468354921 | 6,379869424 | -1,881019106 | 0,004344049 | 0,120651229 | 0 |
|  | HAIRPIN_hsa-mir-4516_at | 5,568515432 | 6,47967804 | -1,880560353 | 0,000697221 | 0,054315223 | 0 |
|  | MATURE_hsa-mir-4508_at | 8,585516683 | 9,484625788 | -1,864914003 | 0,012813836 | 0,231228213 | 0 |
|  | HAIRPIN_hsa-mir-3185_at | 5,637499176 | 6,528858441 | -1,854922955 | 0,003268599 | 0,104399047 | 0 |
|  | MATURE_hsa-mir-4463_at | 6,276352839 | 7,158434111 | -1,843032194 | 0,001261181 | 0,067136855 | 0 |
|  | MATURE_hsa-mir-762_at | 7,922004271 | 8,799357652 | -1,837002234 | 0,004654923 | 0,124994824 | 0 |
|  | MATURE_hsa-mir-4745-5p_at | 6,550635662 | 7,425264098 | -1,833535802 | 0,010751314 | 0,205626923 | 0 |
|  | MATURE_hsa-mir-3620-5p_at | 6,429404865 | 7,289865953 | -1,815618493 | 0,00536871 | 0,135020946 | 0 |
|  | MATURE_hsa-mir-4758-5p_at | 5,276189159 | 6,136259927 | -1,815127345 | 0,000432264 | 0,039447154 | -1 |
|  | MATURE_hsa-mir-371b-5p_at | 4,21417569 | 5,070456496 | -1,810365261 | 0,013164149 | 0,234895483 | 0 |
|  | MATURE_hsa-mir-4739_at | 6,328967436 | 7,177023459 | -1,800073759 | 0,000364039 | 0,038251126 | -1 |
|  | MATURE_hsa-mir-4507_at | 6,653759993 | 7,493325711 | -1,78951138 | 0,008367466 | 0,181325334 | 0 |
|  | HAIRPIN_hsa-mir-4281_at | 6,346237736 | 7,176124184 | -1,77754545 | 0,001763003 | 0,076095032 | 0 |
|  | MATURE_hsa-mir-4281_at | 6,346237736 | 7,176124184 | -1,77754545 | 0,001763003 | 0,076095032 | 0 |
|  | HAIRPIN_hsa-mir-762_at | 7,849927731 | 8,635786345 | -1,724118116 | 0,006871401 | 0,162572255 | 0 |
|  | MATURE_hsa-mir-4492_at | 6,54356067 | 7,323558256 | -1,717128 | 0,034568122 | 0,348298369 | 0 |
|  | MATURE_hsa-mir-4689_at | 5,322936504 | 6,100143434 | -1,71380971 | 0,000459027 | 0,039625181 | -1 |
|  | HAIRPIN_hsa-mir-2861_at | 4,030584349 | 4,790032653 | -1,692843146 | 0,000270742 | 0,038251126 | -1 |
|  | MATURE_hsa-mir-1587_at | 5,339332569 | 6,095826099 | -1,689379594 | 0,009241442 | 0,189883231 | 0 |
|  | MATURE_hsa-mir-3162-5p_at | 4,67566459 | 5,426792105 | -1,683107722 | 0,000662841 | 0,05292782 | 0 |
|  | HAIRPIN_hsa-mir-4508_at | 5,214119609 | 5,960131162 | -1,677149799 | 0,009044212 | 0,187579313 | 0 |
|  | MATURE_hsa-mir-4270_at | 6,509060782 | 7,249952181 | -1,67120811 | 0,001484391 | 0,070043269 | 0 |
|  | HAIRPIN_hsa-mir-4507_at | 5,996786893 | 6,735223164 | -1,668366525 | 0,007221913 | 0,167150659 | 0 |
|  | HAIRPIN_hsa-mir-3665_at | 4,018787391 | 4,743176267 | -1,652200618 | 0,000231302 | 0,038251126 | -1 |
|  | HAIRPIN_hsa-mir-4674_at | 5,315953439 | 6,029778243 | -1,640146636 | 0,003831786 | 0,111717827 | 0 |
|  | HAIRPIN_hsa-mir-4634_at | 4,297892797 | 5,006088406 | -1,633759482 | 0,001693233 | 0,075113687 | 0 |
|  | MATURE_hsa-mir-1910_at | 5,125023452 | 5,824455576 | -1,623865478 | 0,000327171 | 0,038251126 | -1 |
|  | MATURE_hsa-mir-4634_at | 4,215559899 | 4,913290263 | -1,621951144 | 0,00232348 | 0,091756702 | 0 |
|  | MATURE_hsa-mir-4695-5p_at | 5,200441426 | 5,893531994 | -1,616743229 | 0,002500507 | 0,091800205 | 0 |
|  | HAIRPIN_hsa-mir-4741_at | 4,908224649 | 5,559803515 | -1,570886412 | 0,001967624 | 0,079551804 | 0 |
|  | MATURE_hsa-mir-4459_at | 6,035478684 | 6,68209051 | -1,565487324 | 0,001085417 | 0,060821465 | 0 |
|  | HAIRPIN_hsa-mir-4492_at | 6,441609677 | 7,079533929 | -1,556088649 | 0,015074426 | 0,257474416 | 0 |
|  | HAIRPIN_hsa-mir-4505_at | 4,578104864 | 5,19834926 | -1,537135555 | 0,006436608 | 0,154575395 | 0 |
|  | MATURE_hsa-mir-3621_at | 4,836320341 | 5,452352199 | -1,532653804 | 0,011701126 | 0,216947619 | 0 |
|  | MATURE_hsa-mir-572_at | 4,374213351 | 4,988640765 | -1,530950264 | 0,012996364 | 0,233204414 | 0 |
|  | MATURE_hsa-mir-4649-5p_at | 4,71992917 | 5,326810845 | -1,522963826 | 0,004092669 | 0,116714145 | 0 |
|  | HAIRPIN_hsa-mir-1268b_at | 5,938956422 | 6,544096166 | -1,521126087 | 0,002562662 | 0,093012986 | 0 |
|  | HAIRPIN_hsa-mir-4463_at | 5,151710905 | 5,752220685 | -1,516252243 | 0,001922855 | 0,079551804 | 0 |
|  | MATURE_hsa-mir-4532_at | 5,272597942 | 5,858968815 | -1,501465029 | 0,01021411 | 0,198926027 | 0 |
|  | MATURE_hsa-mir-4485_at | 5,267653224 | 4,600638776 | 1,587783763 | 0,030094169 | 0,333352446 | 0 |
|  | HAIRPIN_hsa-mir-155_at | 4,74694122 | 3,892125397 | 1,808527861 | 0,001419094 | 0,070043269 | 0 |
|  | MATURE_hsa-mir-155-5p_at | 4,835147096 | 3,886538428 | 1,930010461 | 0,000945732 | 0,05922877 | 0 |
| VC | MATURE_hsa-mir-150-5p_at | 5,834537711 | 6,505889563 | -1,592564553 | 0,033026036 | 0,68985764 | 0 |
|  | HAIRPIN_hsa-mir-150_at | 5,743057034 | 6,3637229 | -1,537584678 | 0,028495088 | 0,682086879 | 0 |
|  | MATURE_hsa-mir-1202_at | 4,414163888 | 5,027121445 | -1,529391284 | 0,000145306 | 0,061455341 | 0 |
|  | HAIRPIN_hsa-mir-4484_at | 4,541659859 | 3,845286931 | 1,620425764 | 4,34E-06 | 0,004098291 | 1 |
|  | HAIRPIN_hsa-mir-155_at | 5,08046709 | 4,167618656 | 1,882759121 | 0,00111854 | 0,223288566 | 0 |
|  | MATURE_hsa-mir-155-5p_at | 5,18856175 | 4,229253979 | 1,944376727 | 0,000864322 | 0,184042858 | 0 |
|  | MATURE_hsa-mir-4484_at | 6,893061157 | 5,367320213 | 2,879345565 | 5,13E-06 | 0,004098291 | 1 |

*VP, viremic progressors; EC, elite controllers; ART, patients on antiretroviral therapy; HIV-, uninfected donors; VC, viremic controllers; p-val, p-value; q-val, adjusted p-value; rej, rejection value.*
