# Supplementary material for: MicroRNA Profile in CD8+ T-Lymphocytes from HIV-Infected Individuals: Relationship with Antiviral Immune Response and Disease Progression
Source: PLoS One. 2016 May 12;11(5):e0155245. doi: 10.1371/journal.pone.0155245 (PMC4865051; doi:10.1371/journal.pone.0155245)
Supplement: S5 Table — VP, viremic progressors; EC, elite controllers; ART, patients on antiretroviral therapy; HIV-, uninfected donors; VC, viremic controllers. (DOCX) [file pone.0155245.s005.docx]

Supplementary Table 5. Number of predicted gene targets for all the differentially expressed miRNAs in each comparison.

| **resting CD8+ T-cells** | | |
| --- | --- | --- |
| **Comparison** | **Number of differentially expressed miRNAs** | **Number of predicted gene targets** |
| VP vs HIV- | 16 | 2286 |
| EC vs HIV- | 26 | 4051 |
| ART vs HIV- | 52 | 5085 |
| VC vs HIV- | 30 | 5018 |
|  |  |  |
| **stimulated CD8+ T-cells** | | |
| **Comparison** | **Number of differentially expressed miRNAs** | **Number of predicted gene targets** |
| EC vs VP | 29 | 3957 |
| ART vs VP | 10 | 1723 |
| HIV- vs VP | 14 | 2873 |
|  |  |  |
| **stimulated vs resting CD8+ T-cells** | | |
| **Comparison** | **Number of differentially expressed miRNAs** | **Number of predicted gene targets** |
| stimulus vs resting VP | 17 | 1476 |
| stimulus vs resting EC | 22 | 2333 |
| stimulus vs resting ART | 8 | 730 |
| stimulus vs resting HIV- | 83 | 7433 |
| stimulus vs resting VC | 7 | 523 |

*VP, viremic progressors; EC, elite controllers; ART, patients on antiretroviral therapy; HIV-, uninfected donors; VC, viremic controllers.*
